# Perturbing reach elicits anticipatory responses in transport and grasp

**DOI:** 10.3389/fnhum.2024.1423821

**Published:** 2024-10-24

**Authors:** Anna Akbaş, Mariusz P. Furmanek, Sarah Hsu, Mathew Yarossi, Eugene Tunik

**Affiliations:** ^1^Department of Physical Therapy, Movement and Rehabilitation Science, Northeastern University, Boston, MA, United States; ^2^Institute of Sport Sciences, Department of Human Motor Behavior, Academy of Physical Education, Katowice, Poland; ^3^Department of Electrical and Computer Engineering, Northeastern University, Boston, MA, United States; ^4^Department of Physical Therapy, University of Rhode Island, Kingston, RI, United States

**Keywords:** anticipatory adjustments, feedforward control, prehension, reach and grasp coordination, perturbation

## Abstract

**Introduction:**

The purpose of this study was to investigate whether the anticipation of a mechanical perturbation applied to the arm during a reach-to-grasp movement elicits anticipatory adjustments in the reach and grasp components. Additionally, we aimed to evaluate whether anticipatory adjustments in the upper limb might be global or specific to the direction of the perturbation.

**Methods:**

Thirteen healthy participants performed reach-to-grasp with perturbations randomly applied to their dominant limb. Participants were presented with three types of trials: unperturbed (control), trials perturbed in a predictable manner (either Up or Down), or perturbed in a partially predictable manner (knowledge about the perturbation but not its specific direction). EMG activity of 16 muscles, as well as the kinematics of wrist, thumb, and index finger, were acquired and analyzed.

**Results and discussion:**

When the perturbation was expected, EMG activity of the *triceps* and *pectoralis major* muscles significantly increased about 50 – 200 ms before the perturbation onset. Peak acceleration of the reach was significantly higher and occurred earlier relative to control trials. Similar adjustments were observed in the grasp kinematics, reflected as significantly shorter time to peak aperture velocity and acceleration, as well as in increased activity of *flexor* and *extensor digitorum* 100–200 ms before perturbation onset. In summary, our data demonstrate that knowledge of an upcoming perturbation of reach during reach-to-grasp action triggers anticipatory adjustments not only in the muscles controlling the reach component, but also in those controlling grasp. Furthermore, our data revealed that the preparatory activations were generalized, rather than direction specific.

## 1 Introduction

The use of anticipatory strategies to overcome expected perturbations is one of the most remarkable features of human movement coordination. Much of our knowledge about anticipatory control has emanated from researchers studying the control of upright stance, in which anticipatory postural adjustments (APAs) counter self-generated and externally triggered balance disturbances (Belenkiy et al., 1967; Latash et al., 1995; Aruin et al., 2001). APAs are characterized by changes in the activation of the postural muscles that occur ∼100 ms before the onset of the perturbation. It is thought that this pre-movement activation acts to generate forces and moments to counteract those produced from the anticipated perturbation or self-generated movement (Bouisset and Zattara, 1987; Massion, 1992). Conversely, when a perturbation cannot be anticipated individuals are forced to rely on less efficient reactive compensatory strategies (CPAs) to maintain balance (Santos et al., 2010; Aruin et al., 2014). The importance of APAs is highlighted when the ability to produce effective APAs is impaired, as is often observed in older adults (Aruin, 2016), as well as individuals with pathologies such as low back pain (Lomond et al., 2015), stroke (Curuk et al., 2020) or Parkinson’s disease (Jacobs et al., 2009; De Azevedo et al., 2016). For this reason, there has been decades of intensive research into the origins of APAs and their structure, as well as methodologies to improve APAs through intervention.

While the “classical” description of APAs involves preparatory activation of the muscles in the lower limbs, hips and trunk, APAs involving preparatory activity of upper arm for minor, self-perturbations (such as rapid finger flexion) have also been observed (Caronni and Cavallari, 2009; Bruttini et al., 2015). This anticipatory activation of upper limb muscles has been shown to be important for maintaining the segmental stability of the whole arm to preserve the trajectory and accuracy of the reaching or pointing movements (Bonnetblanc et al., 2004; Caronni and Cavallari, 2009; Caronni et al., 2013). Similar to more traditional work on posture and balance, these studies indicate that preparatory activation of proximal segments is used to counteract predicted intersegmental torques generated by perturbations of the distal segment. Therefore, in the present study, we will use the term APAs to refer to anticipatory adjustments of the upper limb posture.

Preparatory responses may also be prominent when the intention of a reaching action is not to point, but to interact with an object, for example reach-to-grasp. Reach-to-grasp action is thought to be comprised of two components - transport of the limb towards the target (transport component) and shaping of the fingers for object interaction (grasp component) (Van Vliet et al., 2013; Furmanek et al., 2019, 2022). These components, although independent, are known to be coordinated in a precise temporal and spatial alignment (Jeannerod, 1981, 1984; Gentilucci et al., 1992; Rand et al., 2006). Compensatory adjustment of one component (i.e. grasp) when the other component (i.e. reach) is unexpectedly disturbed is well characterized (Haggard and Wing, 1991; Rand et al., 2004; Schettino et al., 2017); however, anticipatory adjustments utilized to maintain coordination between reach and grasp in response to perturbation have not been the subject of thorough and rigorous investigation.

Researchers investigating how knowledge of object properties affects reach-to-grasp behavior provide some evidence of anticipatory control (Lukos et al., 2007; Fu et al., 2010). For example, participants instructed to reach and grasp an object with explicit knowledge that its center of mass (COM) is located either to the left, center, or right adjust the placement of their fingers accordingly to prevent the object from rolling. However, when the location of the COM could not be predicted, participants used a ‘default’ distribution of contact points that did not minimize object roll. The observation that upper limb APAs may be “direction specific” with knowledge of the perturbation, and conversely generalized without it, is analogous to the direction dependencies that have been well characterized in standing postural tasks (Piscitelli et al., 2017). However, it remains unclear whether knowledge of forthcoming perturbations of the reaching movement elicit direction specific anticipatory activation of arm muscles to maintain coordination in reach-to-grasp movements. Such knowledge may help to guide interventions for robust recovery from neurological impairment and offer insights into human motor behaviors that may inspire robotic controllers.

In the present study we systematically investigated whether the knowledge of an upcoming mechanical perturbation to the forearm of a reaching limb triggers anticipatory responses both in reach and grasp components. We also test whether those responses are affected by the knowledge of the upcoming perturbation’s directionality (up vs. down) and the explicitness of instruction provided to the participant (knowledge of perturbation vs. knowledge of perturbation and its direction). We hypothesized that the mechanical perturbation applied to the arm during reach-to-grasp action will trigger preparatory responses of muscles activation associated with both transport (reach kinematics) and grasp components (aperture kinematics). We also hypothesized that a general instruction (not direction-specific) will cause generic preparatory responses (e.g., simultaneous activation of agonist-antagonist), while explicit knowledge of the direction of perturbation will elicit direction-specific adjustments (e.g., reciprocal muscle activity) in order to counteract expected direction of perturbation.

## 2 Materials and methods

### 2.1 Participants

Thirteen healthy participants (5F, 8M, age: 24.1 ± 5.7 years, height: 172.7 ± 8.9 cm, body mass: 67.1 ± 12.5 kg, weight of the arm: 3.7 ± 0.9 kg) with normal vision, determined based on self-report during the initial screening session, took part in the study. All participants were informed of the procedures and signed a written informed consent approved by the Northeastern University Institutional Review Board. All procedures conformed to the Declaration of Helsinki (World Medical Association Declaration of Helsinki, 2014). The participants were right-handed based on their own disclosure of preferential hand use for writing, eating, and throwing (Steenhuis and Bryden, 1989) and were free of any orthopedic or neurologic conditions that might interfere with their ability to perform the experiment.

### 2.2 Setup

The laboratory was lit with overhead fluorescent lighting providing a consistent brightness of 500 lux. Position data was acquired using an 8-camera motion tracking system (PPT Studio N™, WorldViz Inc., Santa Barbara, CA, sampling rate: 90 Hz) with IRED markers affixed to the tips of the thumb and index finger, as well as the wrist midway between the ulnar and radial styloid process. Electromyographic activity (EMG) was recorded using a 16-channel Trigno Wireless System (Delsys Inc., MA, USA, sampling rate: 1000 Hz). Skin preparation for reducing impedance was per usual practice. EMG bar electrodes were placed over the muscle bellies, perpendicular to the fibers, of the following muscles on the right side of the body: first dorsal interosseous (FDI), extensor indicis (EI), abductor pollicis brevis (APB), extensor pollicis brevis (EPB), flexor digitorum superficialis (FDS), extensor digitorum communis (EDC), biceps brachii (long head) (BB), triceps brachii (long head) (TB), anterior deltoid (AD), posterior deltoid (PD), upper trapezius (UT), pectoralis major (PEC), latissimus dorsi (LAT), serratus anterior (SER), and bilateral lumbar erector spinae (ESr, ESl). Proper positioning of EMG electrodes was ensured by physically palpating the muscle during sustained isometric contraction and visual confirmation of the EMG signal (Eckerle et al., 2012). A customized MATLAB-based software (MathWorks Inc., R2021b, Natick, MA, USA) was used to acquire and record EMG data.

**FIGURE 1:**
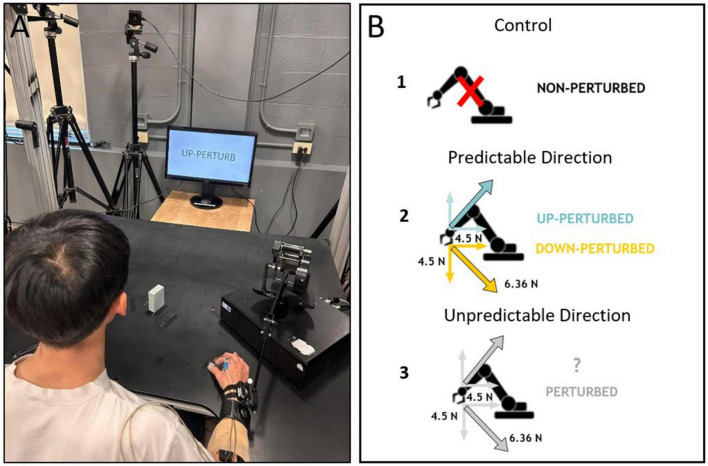
Experimental setup **(A)**, and schematic representation of the experimental conditions **(B)**. B1: “NON-PERTURB” cues indicated the absence of any perturbation. B2: Full information that indicated both the presence, and the direction, of the perturbation. B3: Partial information indicating that there would be a perturbation without specifying the direction. The perturbation component vectors are indicated as gray (when direction was unknown) or colored arrows (when the direction was provided, blue indicates perturbation up and yellow perturbation down). The actual force vector was a resultant vector in either the upward-back or downward-back diagonals.

A Phantom Premium™ haptic device (3D Systems, Inc., Andover, MA) was used to apply a mechanical perturbation to the reaching right limb. The phantom was attached to the participant’s distal forearm 5 cm above the wrist. Perturbations consisted of a ∼ 6.36 N force applied 200 ms after movement onset in one of two diagonal directions: (i) the resultant of two 4.5 N vectors acting either up and towards the participant’s body (referred to as ‘Up’), and (ii) the resultant of two 4.5 N vectors acting either down and towards the participant’s body (referred to as ‘Down’) and was continuously applied until the object was grasped (Figure 1B).

Each participant was instructed to reach, grasp and lift a 3D printed rectangular object (w × h × d: 5.4 × 2.5 × 8.0 cm; 44 g) located 30 cm away from the starting position, along a diagonal axis. The object was rotated (along the long axis) 75 degrees relative to subject’s body to make it biomechanically easier (to avoid wrist extension) to grasp (Figure 1A).

The instruction for each trial was displayed at eye level on a 27-in monitor located 1.8 m in front of the subject. The schedule of trials and triggering of perturbations were controlled using custom-made software developed in C#.

### 2.3 Procedure

Participants were seated on the edge of a height-adjustable stool, with the knees and hips flexed at 90 degrees. Prior to the experiment, each participant performed a maximum voluntary isometric contraction (MVIC) for each muscle (4 s hold). These data were later used for normalization of the respective muscle EMG signal. To begin the experiment, the right hand and forearm were positioned on the table with the thumb and index finger in a pinch position around a 1.5 cm thick wooden peg located 24 cm to the right and 12 cm in front of the body midline (starting position). The thumb depressed a start switch embedded on the thumb-side of the peg, which once released, was used to mark movement onset (Figure 1A). It must be noted that this definition of movement onset does not prevent the observation of deviations in aperture-related kinematics prior to switch release, as in some participants, the index finger could occasionally begin to move before fully disengaging from the switch.

Subjects were instructed to reach, pincer grasp, and lift the object with their dominant right hand without leaning their trunk forward. During reaching participants could experience the perturbation of known direction (Predictable Direction of perturbation−PD), perturbation of unknown direction (Unpredictable Direction of perturbation−UD) or no perturbation (control trials). Before each trial, information about one of four possible forthcoming events was displayed on the screen for 2.5 seconds: (a) ‘non-perturb’, indicating that there would not be a perturbation to the reach (Control, Figure 1B1), (b) ‘up-perturb’, indicating that the perturbation would exert a force on the arm in a diagonal up and back direction (PD-up, Figure 1B2), (c) ‘down-perturb’, indicating that the perturbation would exert a force on the arm in a diagonal down and back direction (PD-down, Figure 1B2), and (d) ‘perturb’, indicating that the perturbation would occur either diagonally up or down (UD-up, UD-down) (Figure 1B3). Participants were asked to initiate reaching movements immediately after the instructions disappeared.

To become familiarized with the experimental set-up and procedure, each participant completed a training protocol that consisted of 40 trials (10 trials per condition in the following order: Control, PD-up, PD-down, and 5 trials per UD-up and UD-down). After familiarization, participants confirmed that they felt comfortable with the task and understood all of the instructions. The experiment began after a 2-min rest.

### 2.4 Experimental design

**FIGURE 2:**
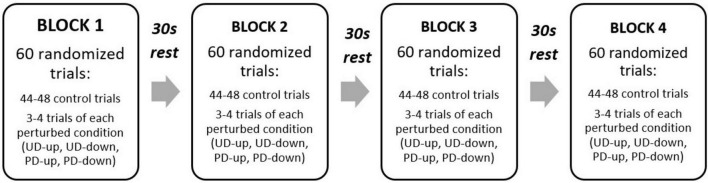
Experimental design. UD, unpredictable direction, PD, predictable direction.

The experiment consisted of 240 trials in total (180 Control, 15 PD-up, 15 PD-down, and 30 perturbed of unknown direction: 15 UD-up and 15 UD-down). To mitigate fatigue and sustain participant engagement, the experiment was divided into 4 blocks with 30 second ‘rest’ periods between each block. To avoid the effect of learning, all trials were presented in a randomized order, counterbalanced across the blocks (Figure 2).

### 2.5 Data analysis

All data analyses were performed in MATLAB (Math Works Inc., R2021b, Natick, MA, USA). Position data were lowpass filtered at 6 Hz with a 4th order Butterworth filter. Grasp- and reach-related variables were calculated based on the markers attached to fingertips and wrist as follows: *Aperture*, as the distance between the fingertip markers; *aperture velocity* and *aperture acceleration*, as the first and second derivative of aperture, respectively; *transport velocity* and *acceleration*, as the first and second derivative of wrist position, respectively. The time to peak aperture / transport velocity and acceleration were calculated as the duration between the time of movement onset (switch release) and each respective peak. When two peaks of velocity appeared during downward perturbation, we always used the first peak, which was consistently larger than the second.

**FIGURE 3:**
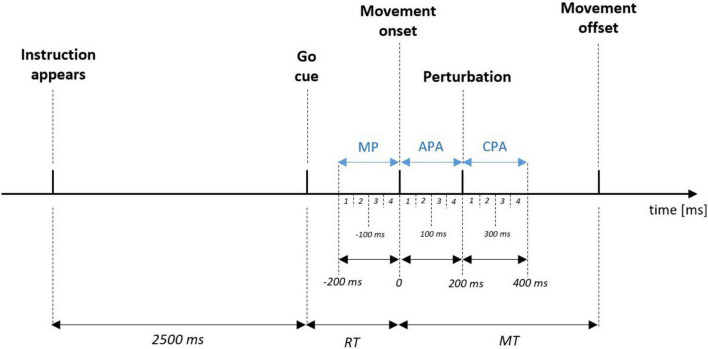
Schematic representation of the order of events and timing of three phases (in blue): up to –200 ms before movement onset—Movement Preparation phase (MP), between movement onset and perturbation - Anticipatory Postural Adjustments phase (APA), and up to 200 ms after perturbation - Compensatory Postural Adjustments phase (CPA). Numbers from 1 to 4 indicate the 50 ms epochs in which integrated muscle activity was calculated. For example, APA4 indicates that the iEMG was calculated in last 50ms (150–200 ms) before the onset of the perturbation. RT, reaction time; MT, movement time.

The EMG signal was first processed by detrending and band-pass filtering (10–300 Hz) using a 4th-order Butterworth filter. The EMG signal was then full wave rectified and smoothed using root mean square at 50 ms epochs, and normalized to the respective muscle’s peak MVIC extracted from the last 3 seconds of the MVIC collection. The integrated muscle activity (iEMG) was calculated for three phases of each trial: (i) Movement Preparation phase (MP), which was the time before switch release; (ii) Anticipatory Postural Adjustment phase (APA), which was the time between movement onset and the perturbation; (iii) Compensatory Postural Adjustment phase (CPA), which was the time after the perturbation. Thus, the iEMG was calculated for the following 50 ms epochs: MP1 (−200 to −151 ms), MP2 (−150–101 ms), MP3 (−100–−51 ms), MP4 (−50–−1 ms), APA1 (0–50 ms), APA2 (51–100 ms), APA3 (101–150 ms), APA4 (151–200 ms), CPA1 (201–250 ms), CPA2 (251–300 ms), CPA3 (301–350 ms), CPA4 (351 – 400 ms), (see also Figure 3).

### 2.6 Statistical analysis

The number of participants in our study was determined by previous research on similar topics, where a comparable number of participants was utilized (Rand et al., 2004; Furmanek et al., 2022).

Paired two-tailed t-tests with Bonferroni correction for multiple comparisons were used to compare kinematics (variables: aperture velocity, aperture, acceleration, transport acceleration) and muscle activity (variables: iEMG at each 50 ms epoch) between non-perturbed (control) and perturbed (UD-up, PD-up, UD-down, PD-down) conditions. The Shapiro-Wilk test was used to verify the normality of data distribution; whenever the assumption was not met, the non-parametric Wilcoxon signed-rank comparison with Bonferroni correction was used.

A 2 × 2 rmANOVA with factors *Direction* (up, down) and *Instruction* (predictable direction, unpredictable direction) was utilized to test for differences in kinematics and muscle activities between directions of perturbation and type of instruction. Given our analysis involved fewer than three factors, Mauchly’s sphericity test was not applicable for detecting violations of sphericity. Significant effects were further explored using two-tailed pairwise comparisons with Bonferroni corrections. Statistical significance was set as alpha = 0.05. All statistical analyses were performed using Statistica v. 13.3 (TIBCO Software Inc.). All variables are presented as means ± 1 standard deviation (SD).

## 3 Results

**FIGURE 4:**
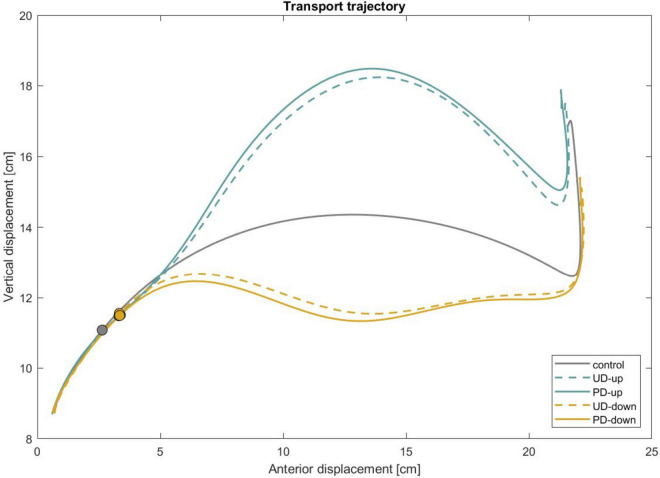
Mean trajectories of transport (wrist marker) across participants are shown under unperturbed conditions (control) as well as under mechanical perturbation with predictable and unpredictable directions. The circle marker indicates the moment in the movement when the perturbation was applied. UD, unpredictable direction; PD, predictable direction.

To assess the potential effect of learning across trials, we compared the first five and last five trials for each condition. Although some performance variation was observed, no significant learning effect emerged. A representative figure and the statistical analysis are included in the Supplementary Table 1 and Supplementary Figure 1. Additionally, we conducted a regression analysis of wrist kinematics across trials, with the results presented in Supplementary Figures 2, 3. All participants completed the task with no report of adverse events. Figure 4 shows the mean trajectories of the transport component across all participants and illustrates the average deviations from the intended reaching path throughout the movement under mechanical perturbation.

### 3.1 Effect of knowledge of perturbation on anticipatory responses

All kinematic data were first tested for normality using the Shapiro-Wilk test, which confirmed normal distribution (*p* > 0.05). No significant outliers were detected in the kinematic variables, and variability across participants was within expected ranges. However, most of the EMG data did not meet normality assumptions, and as a result, non-parametric Wilcoxon signed-rank tests were used for analyzing EMG variables. The results presented here are based on the appropriate statistical tests for each type of data.

#### 3.1.1 Transport component

##### 3.1.1.1 Kinematics

**FIGURE 5:**
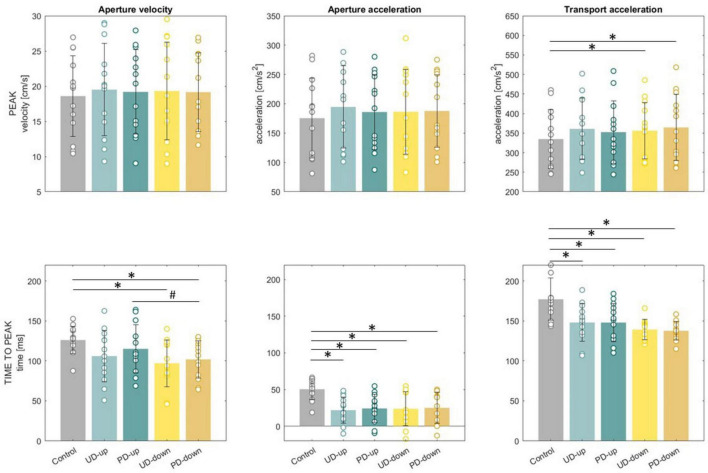
Mean peaks and time to peaks for aperture velocity, aperture acceleration and transport acceleration. Standard deviations showed as error bars. Significant differences between control and perturbed conditions were marked as *. Significant differences between directions of perturbations were marked as #.

A dependent samples t-test revealed that the knowledge of upcoming perturbation significantly influenced transport kinematics. The average peak transport acceleration in perturbed conditions was significantly higher than in non-perturbed condition (Control: 334.8 ± 75.1 cm/s^2^ vs. UD-down: 356.3 ± 71.8 cm/s^2^, t(12) = −4.22, *p* < 0.001; PD-down: 364.5 ± 84.7 cm/s^2^, t(12) = −3.43, *p* < .001), (Figure 5). In perturbed conditions time to peak acceleration was significantly shorter than in non-perturbed condition (Control: 177 ± 26 ms vs. UD-up: 148 ± 23.6 ms, t(12) = 3.39, *p* < 0.05, PD-up: 148 ± 23.9 ms, t(12) = 3.46, *p* < .05, UD-down: 139.2 ± 12.6 ms, t(12) = 4.8, *p* < 0.001, PD-down: 137.6 ± 11.6 ms, t(12) = 5.59, *p* < 0.001), (Figure 5). Figure 5 illustrates the significant differences in transport and grasp kinematics between perturbed and non-perturbed conditions. The earlier time to peak transport acceleration in perturbed conditions supports our hypothesis that the knowledge of an upcoming mechanical perturbation triggers anticipatory responses in the transport component of the movement. All results of dependent sample t-test for kinematics are presented in Supplementary Table 2.

##### 3.1.1.2 Electromyography

**FIGURE 6:**
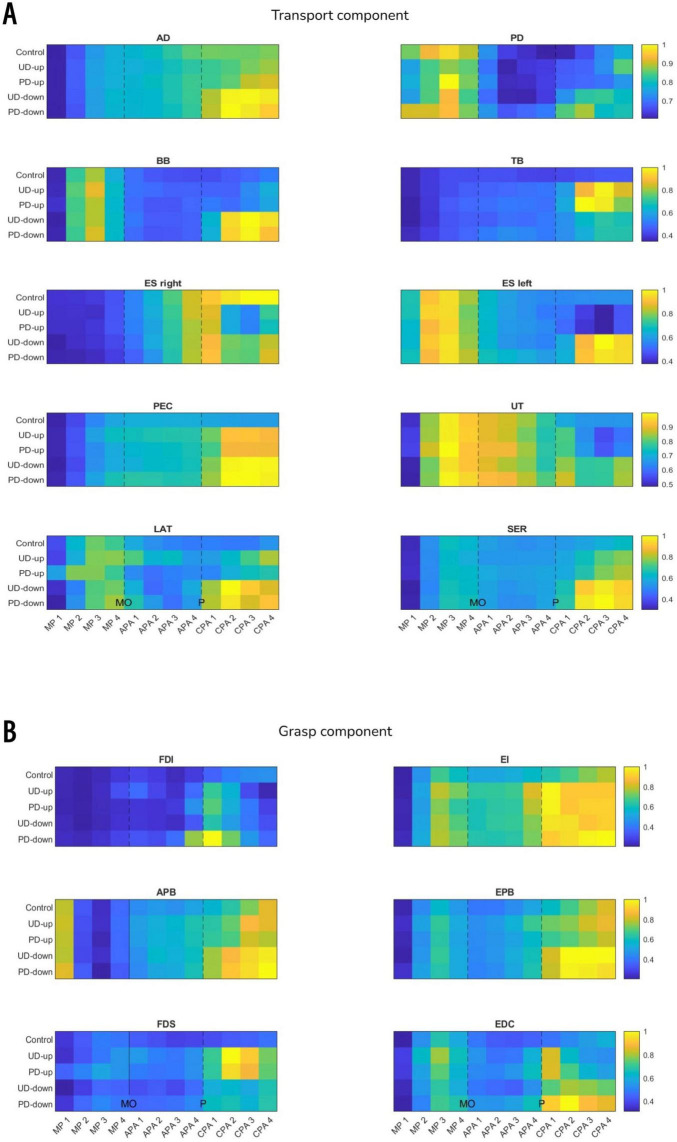
The heatmaps show mean iEMG across all participants in 50 ms time epochs (see Methods) in muscles associated with transport (panel **A**) and grasp (panel **B**) components. Each heatmap was normalized to maximum iEMG across all experimental conditions within a given muscle, therefore 1 indicate maximum, and 0 minimum iEMG of a given muscle (yellow and blue color on heatmap, respectively). For example, the activity of EDC muscle (panel **B**) was the smallest in MP1 phase in all experimental conditions, and the highest in CPA2 phase in PD-down condition. MO, movement onset; P, perturbation; MP, movement preparation; APA, anticipatory postural adjustments; CPA, compensatory postural adjustments.

**FIGURE 7:**
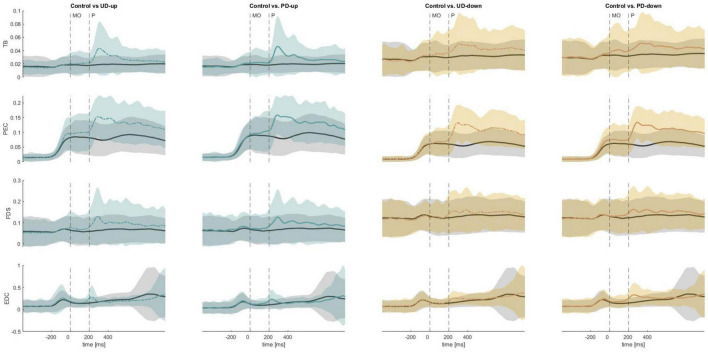
The mean EMG traces across all participants for the triceps brachii (TB), pectoralis major (PEC), flexor digitorum superficialis (FDS), extensor digitorum communis (EDC). The black solid line corresponds to the control condition, whereas the colored lines represent perturbed conditions. MO, movement onset; P, perturbation; UD, unpredictable direction; PD, predictable direction.

A non-parametric Wilcoxon signed-rank comparison showed that the anticipatory iEMG was significantly higher in perturbed conditions in TB (APA4; UD-up: Z = 2.83, *p* = 0.005, UD-down: Z = 2.97, p = 0.003) and PEC (APA1; PD-down: Z = 2.62, *p* = 0.009, APA2; PD-down Z = 2.76, *p* = 0.006, APA3; PD-down: Z = 2.76, *p* = 0.006, APA4; PD-down: Z = 2.83, *p* = 0.005) than in non-perturbed condition (Figure 6A). Figure 7 shows the mean EMG traces for TB and PEC during perturbed and non-perturbed trials. The observed increase in anticipatory muscle activation in the perturbed conditions, aligns with our hypothesis that forearm muscles exhibit increased preparatory activation in anticipation of mechanical perturbations. All results of dependent sample t-test for iEMG are presented in Supplementary Table 3.

#### 3.1.2 Grasp component

##### 3.1.2.1 Kinematics

It was apparent in the finger kinematics several participants utilized a strategy where they initiated index finger extension prior to lifting the thumb off the start switch and advancing the hand toward the object. This shift in strategy was particularly evident in conditions where participants anticipated any form of perturbation (Figure 5). Given our working definition of movement onset as release of the start switch, indicating advancement of the hand towards the object, this can result in a negative value of peak aperture acceleration if participants engaged in this strategy. The use of relative timing in milliseconds to the start switch does not impact use of aperture acceleration as a feature of anticipatory adjustments, nor does it affect statistical procedures as the value used remains in milliseconds and is simply offset by a constant for all values.

A dependent samples t-test revealed that the knowledge of upcoming perturbation applied to forearm significantly influenced grasp kinematics. The average time to peak aperture velocity (126 ± 17 ms) and acceleration (50.4 ± 14.1 ms) in non-perturbed condition occurred significantly later than in time to peak aperture velocity (UD-down: 97 ± 29.2 ms, t(12) = 3.55, *p* < 0.05, PD-down: 101.8 ± 23.3 ms, t(12) = 3.47, *p* < 0.05) and acceleration (UD-up: 21.9 ± 17.9 ms, t(12) = 4.76, *p* < 0.001, PD-up: 24.2 ± 19.2 ms, t(12) = 4.4, *p* < 0.001, UD-down: 23.8 ± 23.3 ms, t(12) = 3.85, *p* < 0.05, PD-down: 25 ± 21.2, ms, t(12) = 3.55, *p* < 0.05) in perturbed conditions (Figure 5). The significant shorter time to peak aperture velocity and acceleration in perturbed conditions, compared to non-perturbed conditions, support our first hypothesis. These findings suggest that knowledge of an upcoming perturbation prompts anticipatory adjustments not only in the reach phase of the movement, but also in grasp. The average peak aperture velocity and peak acceleration did not differ significantly between conditions. All results of dependent sample *t*-test for kinematics are presented in Supplementary Table 2.

##### 3.1.2.2 Electromyography

A non-parametric Wilcoxon signed-rank comparison showed significant increase in anticipatory iEMG in FDS (APA1; UD-up: Z = 2.9, *p* = 0.004, APA2; PD-up: Z = 2.62, *p* = 0.009, APA3; UD-up: Z = 2.76, *p* = 0.006, PD-up: Z = 2.62, *p* = 0.009, APA4; UD-up: Z = 3.04, *p* = 0.002, PD-up: Z = 2.9, *p* = 0.004, PD-down: Z = 2.69, *p* = 0.007) and EDC (APA3; PD-up: Z = 2.69, *p* = 0.007, PD-down: Z = 2.9, *p* = 0.004, APA4; UD-up: Z = 3.11, *p* = 0.002, PD-up: Z = 3.18, *p* = 0.001, UD-down: 2.55, *p* = 0.011, PD-down: Z = 3.18, *p* = 0.001) in perturbed conditions when compared to non-perturbed condition (Figure 6B). The significant increase in anticipatory muscle activation (iEMG) in FDS and EDC under perturbed conditions, compared to non-perturbed trials, further supports our hypothesis that knowledge of the perturbation leads to preparatory responses not only in distal muscles, but also more proximal ones associated with grasp. Figure 7 illustrates the increased mean EMG traces for the FDS and EDC muscles across participants prior to the onset of the perturbation, particularly in the perturbed conditions compared to the control condition. No difference was found in anticipatory iEMG in FDI, EI, APB and EPB. All results of dependent sample t-test for iEMG are presented in Supplementary Table 3.

### 3.2 Effect of knowledge of direction of perturbation and instruction on anticipatory responses

#### 3.2.1 Transport component

##### 3.2.1.1 Kinematics

A 2 × 2 rmANOVA revealed a significant main effect of *Direction* for time to peak transport acceleration (F_(1,12)_ = 7.68, *p* = 0.017), suggesting that knowledge of the perturbation’s direction influences the temporal dynamics of transport. However post-hoc analyses did not show significant differences, indicating that although the direction of perturbation had an overall effect, the specific directional contrasts may be more subtle. There were no significant main effects of *Instruction* or interaction between factors *Direction and Instruction* for peak transport acceleration. These findings suggest that while the participants were able to anticipate the perturbation, the detailed nature of that anticipation may not differ substantially across instructional conditions, a result that partly supports our hypothesis of direction-specific adjustments. All results of rmANOVA for kinematics are presented in Supplementary Table 4.

##### 3.2.1.2 Electromyography

A 2 × 2 rmANOVA showed significant main effect of *Direction* for right ES (MP1: F_(1,12)_ = 6.275, *p* = 0.028, η^2^ = 0.343) and SER (MP3: F_(1,12)_ = 5.292, *p* = 0.04, η^2^ = 0.305). The significant effect of *Instruction* was found in PD (APA3: F_(1,12)_ = 4.82, *p* = 0.049, η^2^ = 0.286), BB (APA2: F_(1,12)_ = 6.64, *p* = 0.024, η^2^ = 0.356), left ES (MP2: F_(1,12)_ = 5.55, *p* = 0.036, η^2^ = 0.316) and UT (APA2: F_(1,12)_ = 6.31, *p* = 0.027, η^2^ = 0.345). There was a significant interaction between factors *Direction and Instruction* for PEC (MP3: F_(1,12)_ = 8.022, *p* = 0.015, η^2^ = 0.401; MP4: F_(1,12)_ = 14.518, *p* = 0.002, η^2^ = 0.547; APA1: F_(1,12)_ = 8.976, *p* = 0.011, η^2^ = 0.428; APA2: F_(1,12)_ = 9.92, *p* = 0.008, η^2^ = 0.453; APA3: F_(1,12)_ = 5.689, *p* = 0.034, η^2^ = 0.322), UT (MP4: F_(1,12)_ = 6.314, *p* = 0.027, η^2^ = 0.345) and SER (APA1: F_(1,12)_ = 6.047, *p* = 0.03, η^2^ = 0.335) (Figure 6A). Despite these significant differences, the pattern of EMG responses did not consistently align with the hypothesized direction-specific adjustments. Thus, we cannot conclude that the anticipated effects based on directionality and explicit instruction were uniformly observed across all conditions. The post-hoc results are presented in Table 1. All results of rmANOVA for iEMG are presented in Supplementary Table 5.

**TABLE 1:** The results of post-hoc analysis for significant main effect *Direction* and interaction between *Direction and Instruction*.

Msl	Condition		MP 1	MP 2	MP 3	MP 4	APA 1	APA 2	APA 3	APA 4	CPA 1	CPA 2	CPA 3	CPA 4
**Transport component**														
**AD**	Up vs. Down	UD	—	—	—	—	—	—	—	—	**0.005**	**<0.001**	**<0.001**	**<0.001**
		PD	—	—	—	—	—	—	—	—	**0.002**	**<0.001**	**<0.001**	0.026
**PD**	Up vs. Down	UD	—	—	—	—	—	—	—	—	0.041	0.057	—	—
		PD	—	—	—	—	—	—	—	—	0.045	**0.010**	—	—
**BB**	Up vs. Down	UD	—	—	—	—	—	—	—	—	**0.008**	**0.002**	**0.003**	**0.010**
		PD	—	—	—	—	—	—	—	—	**0.002**	**0.001**	**0.012**	**0.016**
**TB**	Up vs. Down	UD	—	—	—	—	—	—	—	—	—	**0.018**	**0.014**	**0.023**
		PD	—	—	—	—	—	—	—	—	—	0.032	0.025	0.030
**ESr**	Up vs. Down	UD	0.037	—	—	—	—	—	—	—	—	**<0.001**	**<0.001**	—
		PD	0.195	—	—	—	—	—	—	—	—	**0.004**	**0.001**	—
**ESl**	Up vs. Down	UD	—	—	—	—	—	—	—	—	**0.003**	**<0.001**	**<0.001**	**<0.001**
		PD	—	—	—	—	—	—	—	—	**0.011**	**<0.001**	**<0.001**	**<0.001**
**PEC**	Up vs. Down	UD	—	—	0.098	**0.014**	0.078	0.049	**0.013**	—	—	**0.012**	**0.020**	**<0.001**
		PD	—	—	0.075	**0.012**	0.069	0.043	0.298	—	—	**0.008**	0.026	**0.023**
**UT**	Up vs. Down	UD	—	—	—	0.294	—	—	—	—	**0.012**	**0.004**	**0.001**	**0.008**
		PD	—	—	—	0.135	—	—	—	—	0.033	0.161	**0.020**	**0.006**
**LAT**	Up vs. Down	UD	—	—	—	—	—	—	—	—	—	**0.003**	**0.001**	0.493
		PD	—	—	—	—	—	—	—	—	—	**0.010**	**0.008**	**0.012**
**SER**	Up vs. Down	UD	—	—	0.316	—	0.027	—	—	—	**<0.001**	**<0.001**	**0.005**	**0.005**
		PD	—	—	0.301	—	0.231	—	—	—	**0.013**	**0.001**	**0.004**	**0.015**
**Grasp component**														
**FDI**	Up vs. Down	UD	—	—	—	—	—	—	—	—	—	—	—	—
		PD	—	—	—	—	—	—	—	—	—	—	—	—
**EI**	Up vs. Down	UD	—	—	—	**0.009**	—	—	—	—	—	—	—	—
		PD	—	—	—	0.029	—	—	—	—	—	—	—	—
**APB**	Up vs. Down	UD	—	—	—	—	—	—	—	—	—	—	—	—
		PD	—	—	—	—	—	—	—	—	—	—	—	—
**EPB**	Up vs. Down	UD	—	—	—	—	—	—	—	—	0.064	0.027	0.040	0.062
		PD	—	—	—	—	—	—	—	—	**0.003**	**0.013**	0.026	0.052
**FDS**	Up vs. Down	UD	—	—	—	—	—	—	—	0.038	—	0.032	0.027	—
		PD	—	—	—	—	—	—	—	0.459	—	0.068	0.066	—
**EDC**	Up vs. Down	UD	—	—	—	0.049	—	—	—	—	—	—	**0.003**	**0.001**
		PD	—	—	—	0.108	—	—	—	—	—	—	**0.002**	**<0.001**

Bold *p*-values indicate significant differences between directions after Bonferroni correction for multiple comparison. MP, movement preparation phase; APA, anticipatory postural adjustment phase; CPA, compensatory postural adjustment phase; UD, unpredictable direction; PD, predictable direction; AD, anterior deltoid; PD, posterior deltoid; UT, upper trapezius; PEC, pectoralis major; LAT, latissimus dorsi; SER, serratus anterior; ESr, lumbar erector spinae right; ESl, lumbar erector spinae right; FDI- first dorsal interosseous; EI - extensor indicis; APB, abductor pollicis brevis; EPB, extensor pollicis brevis; FDS, flexor digitorum superficialis; EDC, extensor digitorum communis; BB, biceps brachii (long head); TB, triceps brachii (long head). The table shows the differences in iEMG between perturbation up and perturbation down in two instruction types (Unpredictable Direction and Predictable Direction).

#### 3.2.2 Grasp component

##### 3.2.2.1 Kinematics

A 2 × 2 rmANOVA revealed a significant main effect of *Direction* for time to peak aperture velocity (F_(1,12)_ = 11.71, *p* = 0.005), with post-hoc tests showing significant differences between PD-up and PD-down conditions (*p* = 0.009), (Figure 5). However, there were no significant main effects of *Direction* for peak aperture velocity, as well as peak and time to aperture acceleration. Similarly, no significant effects of Instruction or interaction between factors Direction and Instruction were observed for any of the analyzed grasp-related variables, suggesting that the hypothesized direction-specific and instruction-based anticipatory adjustments in grasp kinematics were not confirmed. All results of rmANOVA for kinematics are presented in Supplementary Table 4.

##### 3.2.2.2 Electromyography

A 2 × 2 rmANOVA revealed a significant main effect of *Direction* for FDS in APA4 (F_(1,12)_ = 5.531, *p* = 0.037, η^2^ = 0.316). There was a significant interaction between factors *Direction and Instruction* for EI in MP4 (F_(1,12)_ = 6.22, *p* = 0.028, η^2^ = 0.341) and EDC in MP4 (F_(1,12)_ = 5.078, *p* = 0.044, η^2^ = 0.297) (Figure 6B). However, there were no significant main effects of *Instruction* on anticipatory responses in iEMG responses in other muscles. These findings indicate that while certain muscles showed direction- and instruction-related effects, the overall hypothesis regarding anticipatory EMG responses based on explicit directional knowledge was not fully supported. The post-hoc results are presented in Table 1. All results of rmANOVA for iEMG are presented in Supplementary Table 5.

### 3.3 Effect of direction of perturbation and instruction on compensatory responses

#### 3.3.1 Transport component

As expected, *Direction* of perturbation significantly influenced compensatory responses in all of the muscles associated with transport component. The significant effect of *Instruction* was found only in BB in CPA 4 phase. The significant effect of interaction was found in AD in CPA 4 phase. Significant main effects are summarized in Table 2, while the results of post-hoc are presented in Table 1.

**TABLE 2:** Significant main effects of rmANOVA with factors of Direction, Instruction and interaction between Direction and Instruction for muscles associated with transport component in compensatory postural adjustments phase in each of four 50 ms epochs (CPA 1–4).

Muscle	Main effect	CPA 1			CPA 2			CPA 3			CPA 4		
		**F_(1,12)_**	***p***	**η ^2^**	**F_(1,12)_**	***p***	**η ^2^**	**F_(1,12)_**	***p***	**η ^2^**	**F_(1,12)_**	***p***	**η ^2^**
**AD**	Direction	17.89	0.001	0.6	41.16	<0.001	0.77	57.2	<0.001	0.83	23.43	<0.001	0.66
	Instruction	—	—	—	—	—	—	—	—	—	—	—	—
	Dir x Instr	—	—	—	—	—	—	16.58	0.002	0.58	6.93	0.022	0.36
**PD**	Direction	7.74	0.017	0.39	12.95	0.017	0.52	—	—	—	—	—	—
	Instruction	—	—	—	—	—	—	—	—	—	—	—	—
	Dir x Instr	—	—	—	—	—	—	—	—	—	—	—	—
**BB**	Direction	16.3	0.002	0.58	8.37	0.001	0.6	10.93	0.006	0.48	8.74	0.012	0.042
	Instruction	—	—	—	—	—	—	—	—	—	—	—	—
	Dir x Instr	—	—	—	—	—	—	—	—	—	—	—	—
**TB**	Direction	—	—	—	6.89	0.022	0.36	7.52	0.018	0.39	6.96	0.022	0.37
	Instruction	—	—	—	—	—	—	—	—	—	10.42	0.007	0.46
	Dir x Instr	—	—	—	—	—	—	—	—	—	—	—	—
**ESr**	Direction	—	—	—	18.98	0.001	0.61	26.87	0.001	0.69	—	—	—
	Instruction	—	—	—	—	—	—	—	—	—	—	—	—
	Dir x Instr	—	—	—	—	—	—	—	—	—	—	—	—
**ESl**	Direction	11.57	0.005	0.49	33.21	0.001	0.73	58.26	0.001	0.83	38.65	0.001	0.76
	Instruction	—	—	—	—	—	—	—	—	—	—	—	—
	Dir x Instr	—	—	—	—	—	—	—	—	—	—	—	—
**PEC**	Direction	—	—	—	10.83	0.006	0.47	8.36	0.014	0.41	3.34	0.003	0.53
	Instruction	—	—	—	—	—	—	—	—	—	—	—	—
	Dir x Instr	—	—	—	—	—	—	—	—	—	—	—	—
**UT**	Direction	10.7	0.07	0.47	5.04	0.04	0.3	12.14	0.05	0.5	10.73	0.007	0.47
	Instruction	—	—	—	—	—	—	—	—	—	—	—	—
	Dir x Instr	—	—	—	—	—	—	—	—	—	—	—	—
**LAT**	Direction	—	—	—	11.6	0.005	0.49	19.54	0.001	0.62	8.58	0.013	0.42
	Instruction	—	—	—	—	—	—	—	—	—	—	—	—
	Dir x Instr	—	—	—	—	—	—	—	—	—	—	—	—
**SER**	Direction	14.95	0.002	0.55	20.78	0.001	0.63	12.85	0.004	0.52	11.61	0.005	0.49
	Instruction	—	—	—	—	—	—	—	—	—	—	—	—
	Dir x Instr	—	—	—	—	—	—	—	—	—	—	—	—

CPA, compensatory postural adjustment phase; AD, anterior deltoid; PD, posterior deltoid; UT, upper trapezius; PEC, pectoralis major; LAT, latissimus dorsi; SER, serratus anterior; ESr, lumbar erector spinae right; ESl, lumbar erector spinae right; FDI, first dorsal interosseous; EI, extensor indicis; APB, abductor pollicis brevis; EPB, extensor pollicis brevis; FDS, flexor digitorum superficialis; EDC, extensor digitorum communis; BB, biceps brachii (long head); TB, triceps brachii (long head).

#### 3.3.2 Grasp component

*Direction* of perturbation significantly influenced compensatory response of the muscles associated with grasp component: EPB: (CPA1: F_(1,12)_ = 12.33, *p* = 0.004, η^2^ = 0.51, CPA2: F_(1,12)_ = 7,90, *p* = 0.016, η^2^ = 0.4, CPA3: F_(1,12)_ = 7.06, *p* = 0.021, η^2^ = 0.37, CPA4: F_(1,12)_ = 5, *p* = 0.046, η^2^ = 0.29); FDS (CPA2: F_(1,12)_ = 5.06, *p* = 0.044, η^2^ = 0.3, CPA3: F_(1,12)_ = 5.34, *p* = 0.039, η^2^ = 0.31); EDC (CPA3: F_(1,12)_ = 15.67, *p* = 0.002, η^2^ = 0.7, CPA4: F_(1,12)_ = 22.84, *p* < 0.001, η^2^ = 0.66). There was also significant main effect of interaction between Direction and Instruction on EDC in CPA 4 phase (F_(1,12)_ = 8.1, *p* = 0.025, η^2^ = 0.4). The post-hoc results are presented in Table 1. There was no significant effect of *Instruction* on compensatory responses in grasp.

## 4 Discussion

In this study, we investigated whether the anticipation of a mechanical perturbation applied to the arm during a reach-to-grasp movement leads to anticipatory adjustments in the reach and grasp components. We also tested how the explicitness of instructions and the knowledge of direction of the perturbation influence anticipatory responses. To address these questions, participants were instructed to perform reach-to-grasp movements while their reaching limb experienced different conditions: unperturbed, perturbed in a predictable manner (either Up or Down), or perturbed in a partially predictable manner (knowledge about the perturbation but not its specific direction). Our findings revealed that the knowledge of an upcoming forearm perturbation triggered anticipatory adjustments not only in the muscles controlling the transport component of the movement but also in those responsible for the grasp component, despite no actual perturbation occurring in the grasp component. Furthermore, our data revealed that the preparatory activations were generalized, regardless of the specificity of instruction and the direction of the perturbation.

### 4.1 Knowledge of perturbation triggers anticipatory responses in reach and grasp

We observed anticipatory behaviors in the trials where the information about the upcoming perturbation was available. When expecting perturbation, participants significantly increased TB and PEC muscle activation before the onset of the perturbation (Figure 6A) resulting in significantly greater reach acceleration in a shorter period of time (Figure 5) compared to control trials. The pattern of preparatory muscle activation can be explained by considering two important task parameters: the location of the object being grasped, and the direction of the force field exerted by the robotic arm. To grasp an object, participants had to reach with their arm forward and towards the midline of the body, while overcoming the force field acting in combined directions, either backward and upwards or backward and downwards. It is likely, that early activation of TB, the primary extensor of elbow joint, and PEC, strong adductor and internal rotator of the arm and the assistant in the flexion of the arm, were coordinated in feedforward manner to exert the force in forward direction that would overcome the backward perturbation. This observation corroborates previous findings by Button et al. (2002) who observed greater velocities of the reaching limb during an interception task when subjects were expecting mechanical perturbation compared to non-perturbed trials. Noteworthy, the tendency to accelerate motor actions in advance to expected perturbation applies to various types of perturbations, not only the mechanical (proprioceptive) ones. For example, when participants were expecting visual occlusion during catching action, the time to peak reach velocity was reached earlier and the forward displacement of the reach was increased no matter if the time of perturbation was predictable or unpredictable (Tijtgat et al., 2011). The collective findings suggest a general strategy employed to address perturbations occurring late in a movement, typically after reaching peak velocity or acceleration when the limb’s inertia is increased.

Interestingly, our data show that the knowledge of a mechanical perturbation applied to the forearm during a reaching movement elicited preparatory responses not only in transport (increased reach peak acceleration), but also grasp (shorter time to peak aperture velocity and acceleration) (Figure 5), despite no perturbation applied directly to the grasp component. Interpreting these data within the framework of coordinated spatial and temporal reach-to-grasp control, the early compensation observed in the grasp component could be attributed to the initially increased arm acceleration. The sudden spatial change in limb position prompted the hand to open faster, thereby maintaining the stereotypical relationship between both components. Previous findings confirm that perturbation of one component (reach or grasp) can cause compensatory modifications in the other. For instance, investigations utilizing perturbations in object size or position have shown that changes in the grasp component lead to adjustments in limb transport (Bootsma and van Wieringen, 1992; Coats et al., 2008). Similar effects have been observed when participants were required to make rapid online corrections in response to mechanical perturbations applied during transport (Haggard and Wing, 1991; Schettino et al., 2017). Unique to our study, the adjustments in grasp were observed *before the onset* of the perturbation in transport, indicating that the control of both grasp and transport is mediated not only by reactive (feedback) mechanisms but also by predictive (feedforward) programming. Previous findings support feedforward programming of reach-to-grasp (Hoff and Arbib, 1993; Ulloa and Bullock, 2003; Rand et al., 2004), however to the best of our knowledge the only study to suggest anticipatory responses in grasp to a reach perturbation was that of Rand et al. (2004). In that study the authors suggest that co-adaptative changes in hand opening and hand transport in response to an external perturbation of transport (using an elastic band) are the result of both feedforward (anticipatory) and feedback (reactive) control mechanisms. Primary evidence of anticipatory control was the presence of aftereffects when the perturbation was removed. However, omission of EMG measurement and use of constant perturbation in the form an elastic band did not allow Rand’s team for direct measurement of the anticipatory response.

In the present study the anticipatory responses were directly observed in the antagonistic pair of finger muscles (FDS and EDC), but not in the muscles directly controlling pincer grasp (flexors and extensors of index: FDI, EI) and thumb: APB, EPB), (Figure 6B). Increasing the joints stiffness and stability by simultaneous contraction of agonist and antagonist, i.e. co-contraction, is known strategy of the central nervous system (CNS) to maintain control of voluntary movements (De Serres and Milner, 1991). In contrast to depending solely on feedback control, a common strategy to alleviate the negative impacts of perturbations on movement outcomes involves proactively stiffening joints in a feedforward manner. This approach compensates for the typical delay observed in reactions driven by feedback (Jacks et al., 1988). We suppose that preparatory activity of FDS and EDC muscles was increased in order to enhance the rigidity of the joints connecting the low mass segments of the fingers, which are particularly susceptible to external force disturbances. As a result, the stiffened middle, ring, and little fingers could establish a steady base for gripping actions performed by the remaining digits, i.e. the index and thumb. It is worth mentioning that both the FDS and EDC muscles cross the wrist joint, suggesting their potential involvement not only in stabilizing the fingers but also in stabilizing the wrist of the reaching limb. However, since we did not measure the activity of the primary stabilizers of the wrist, we are unable to verify this hypothesis.

The question of why co-contraction was not observed in antagonistic muscle pairs controlling index and thumb should also be addressed. According to previous findings, when individuals are aware of an upcoming perturbation, it is expected that they exhibit either anticipatory co-contraction of muscles or direction-specific muscle activation (Aruin and Latash, 1995; Piscitelli et al., 2017; Forman et al., 2020). However, neither of these responses were observed in the muscles involved in grip control. Considering that increased co-contraction can impede movement execution, it is likely that the CNS intentionally avoided increasing joint stiffness to facilitate the opening of the grip aperture, and compensatory muscle activation was utilized to counteract the force perturbation instead (Santos et al., 2010). Similar finding were observed by Forman et al. (2020) who demonstrated that reduced co-contraction levels were associated with greater maximum angular displacement of the wrist during dynamic wrist tracking task and supporting the notion that decreased co-contraction enhances task performance.

### 4.2 Instruction and direction did not substantially affect anticipatory responses

Despite observing statistically significant effects of *Instruction* on several muscles’ preparatory responses, our overall findings did not support the hypothesis that the explicit or partial knowledge of upcoming perturbations would elicit instruction-specific preparation in grasp or transport (Table 1). We also did not reveal clear effects of *Direction* on the preparation for upward or downward perturbations (Table 1). We hypothesized that general instruction about the upcoming perturbation, without specifying its direction, would result in generic preparatory responses like muscle co-contraction. We also expected that knowing the direction of perturbation would trigger direction-specific anticipatory responses, including reciprocal muscle activation and adjusted reach trajectory. However, we did observe significant differences in the compensatory control of the muscles involved in both transport (all analyzed muscles) and grasp (EPB and EDC) between upward and downward perturbations (Table 1).

Our hypotheses were formulated based on previous studies examining anticipatory postural control, including research conducted by Latash et al. (1995), Santos and Aruin (2008), Leonard et al. (2011), and Piscitelli et al. (2017). For example, Piscitelli et al. (2017) observed a significant prevalence of co-contraction and delayed onset of anticipatory postural adjustments (APAs) when participants were unable to predict the direction of upcoming load perturbations. In contrast, when the direction of the perturbation could be anticipated, the authors observed a reciprocal pattern of muscle activity, indicating the need for compensation specific to the anticipated direction of the perturbation. Similarly, in the study by Leonard et al. (2011), the researchers investigated whether postural adjustments in the legs, necessary for making online corrections to arm movements, were predictive or driven by feedback from the moving limb. Their findings consistently demonstrated that corrections of arm movements in response to target displacement during stance were always preceded by APAs in the leg opposite to the direction of the target shift, providing further support for the directional specificity of APAs.

Our findings differ from previous studies, and we attribute this difference to multiple factors. Firstly, the relatively small perturbation force in our study (∼ 6.36 N) compared to the much higher forces used in studies by Piscitelli et al. (2017) and Rand et al. (2004) (almost 50 N and 33 N, respectively) could explain why we did not observe instruction-specific preparatory responses. Additionally, the stable sitting position maintained by participants during our study may have contributed to the absence of such responses. Another perspective to consider is that the participants focused predominantly on countering the perturbation directed back towards them rather than differentiating between upward and downward directions, despite the distinct perception of upward and downward perturbations. This directional consistency in their strategy might have influenced the lack of significant differences observed in some variables between upward and downward trials. Altogether, our results indicate that the CNS avoided preparatory responses and instead relied on online corrections to counteract perturbations in various directions. This preference aligns with the recognized high metabolic costs associated with anticipatory strategies (Hogan, 1984; Osu et al., 2009). The adoption of an impedance control strategy suggests that the CNS prioritizes movement optimization by balancing necessary neural activity while minimizing energy expenditure, leading to a potential abandonment of anticipatory control in favor of post-perturbation correction (Burdet et al., 2001; Osu et al., 2009; Major et al., 2018). Given the seemingly simplistic nature of the task in our study, the utilization of anticipatory control by the CNS may have been inefficient and unnecessary. We are aware that our study has several limitations. Firstly, as mentioned earlier, the low force perturbations used, along with the participants’ stable sitting position, may not have been sufficiently challenging to elicit instruction and direction-specific anticipatory responses. In future studies, it would be beneficial to consider implementing higher force perturbations or introducing more dynamic and unpredictable conditions to further investigate anticipatory mechanisms. Secondly, expanding the analysis and including wrist muscles could provide valuable insights into upper limb dynamics in response to mechanical perturbation. Moreover, examining other factors that might influence anticipatory responses, such as cognitive factors or task complexity, could enhance our understanding of the underlying mechanisms involved.

Another potential limitation of the study is the use of the thumb press to initiate the task, which may have influenced the coordination of reach and grasp. Participants had to extend their fingers to release the start switch, potentially affecting their overall strategy. Although the force exerted on the start switch was minimal (less than 1N), this setup element, along with the starting position, could have impacted the task performance.

While our study provided valuable insights into APAs during reach-to-grasp tasks, several avenues remain for future research. Firstly, incorporating grasp force measurements could enrich our understanding of how grasping dynamics are affected by perturbations. Although our current focus was on EMG signals and position data to investigate APAs, integrating grasp force data could provide a more comprehensive view of CPAs and their interaction with APAs. Secondly, the variability in terminal position height observed in our study raises intriguing questions about control strategies and anticipatory actions under different perturbation conditions. Future studies should explore the impact of finger placement and object height on wrist kinematics and muscle control, potentially requiring a controlled final position to assess these factors more systematically. Lastly, the rich EMG data collected in this study presents an opportunity to employ advanced EMG analysis methods, such as muscle synergy analysis, to uncover more nuanced insights into muscle coordination during anticipatory adjustments.

In summary, the primary finding of this study was that knowledge of an upcoming forearm perturbation triggered anticipatory adjustments not only in the muscles controlling the transport component of the movement but also in those controlling the grasp component. Furthermore, we revealed that the preparatory activations were generalized, regardless of the specificity of the instruction and the direction of the perturbation. These findings support previous research indicating that the reach and grasp components are highly coordinated not only during movement execution, but critically during reach-to-grasp movement planning in conditions when a perturbation of limb state needs to be overcome. The obtained knowledge has the potential to be translated into the rehabilitation of upper limb recovery in patients with impaired anticipatory control arising from neurological conditions. Additionally, it has the potential to inspire the development of robotic controllers designed to assist and enhance therapeutic interventions.

## Data Availability

The raw data supporting the conclusions of this article will be made available by the authors, without undue reservation.
