# Supplementary material for: Perturbing reach elicits anticipatory responses in transport and grasp
Source: Front Hum Neurosci. 2024 Oct 24;18:1423821. doi: 10.3389/fnhum.2024.1423821 (PMC11540710; doi:10.3389/fnhum.2024.1423821)
Supplement: Supplementary file 1 [file Data_Sheet_1.docx]

**Table S1.** Results of Paired T-Test and Wilcoxon Signed-Rank Test for Wrist Kinematics, Comparing the First and Last 5 Trials.

| **Condition** | | **Mean ± SD (First 5)** | **Mean ± SD (Last 5)** | **t / Z*** | **p-value** |
| --- | --- | --- | --- | --- | --- |
| **Peak Wrist Velocity** | | | | | |
|  | UD-up | 63.36 ± 7.65 | 62.83 ± 9.30 | 0.32 | 0.753 |
|  | PD-up | 63.29 ± 7.47 | 62.41 ± 10.64 | 0.51 | 0.617 |
|  | UD-down | 54.81 ± 10.11 | 52.68 ± 10.45 | 1.72 | 0.11 |
|  | PD-down | 53.23 ± 10.41 | 55.17 ± 11.36 | -1.3 | 0.22 |
| **Time to Peak Wrist Velocity** | | | | | |
|  | UD-up | 29.71 ± 4.27 | 29.97 ± 3.98 | -0.24 | 0.813 |
|  | PD-up | 29.63 ± 3.22 | 31.00 ± 4.87 | -1.05 | 0.312 |
|  | UD-down | 24.48 ± 2.52 | 23.80 ± 2.38 | 1.34 | 0.205 |
|  | PD-down | 24.31 ± 3.84 | 24.71 ± 2.10 | 1.29* | 0.196 |


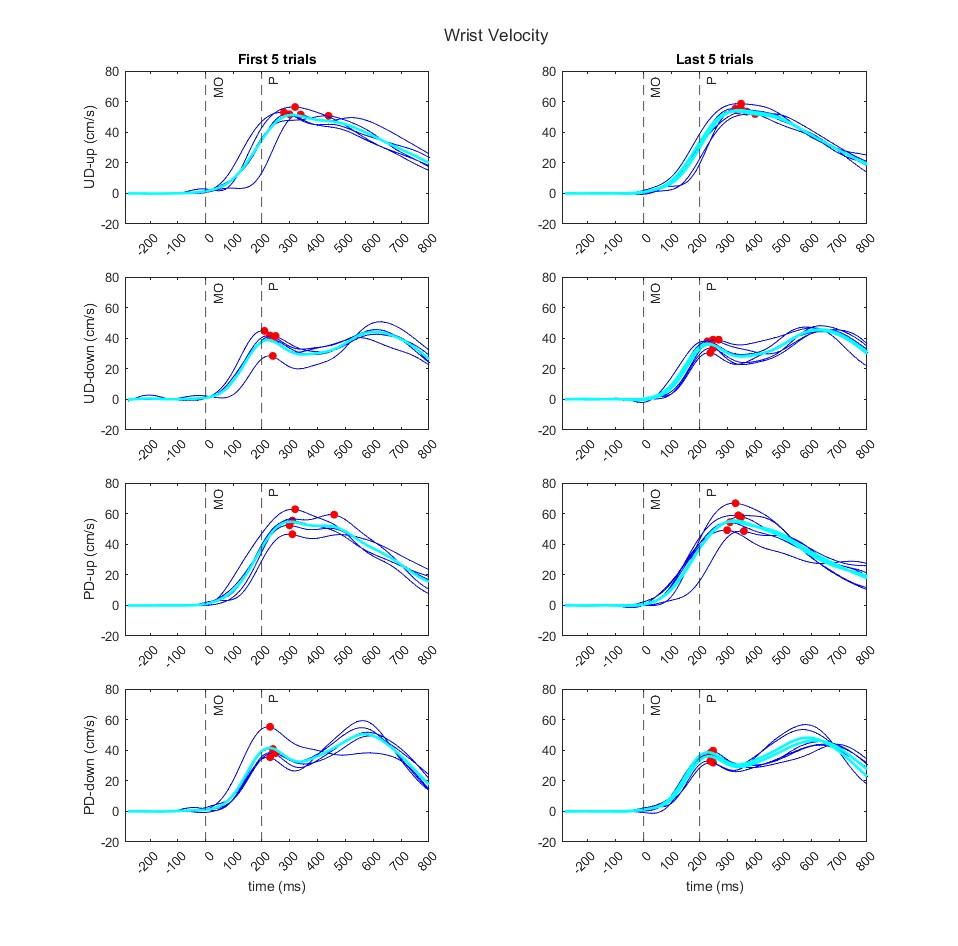


**Figure S1.** Wrist kinematics comparison between the first 5 and last 5 trials. The graph illustrates wrist velocity for a representative participant, marking movement onset (MO), perturbation onset (P), and velocity peaks (red dots).

**
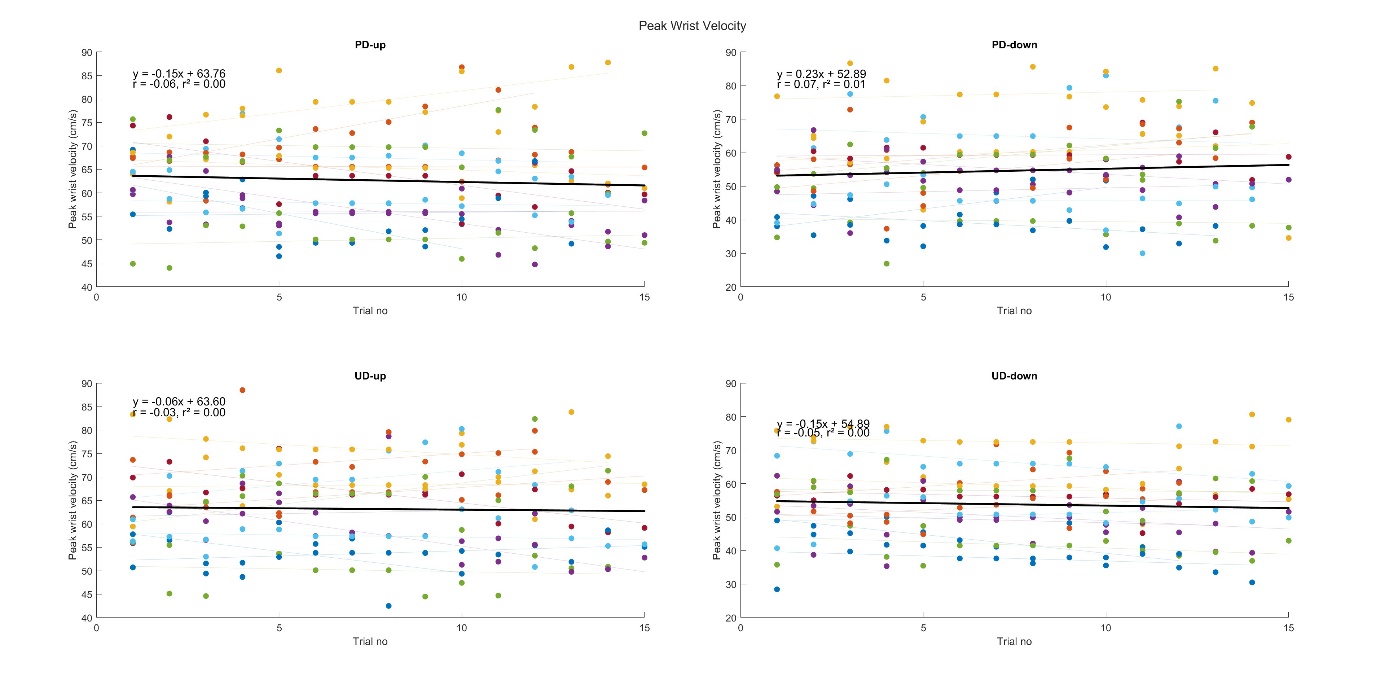
Figure S2.** Regression analysis of wrist kinematics across trials. Peak wrist velocity as a function of trial number, with individual data points color-coded by participant. The bold black line represents the group-level regression, indicating the overall trend across all participants.


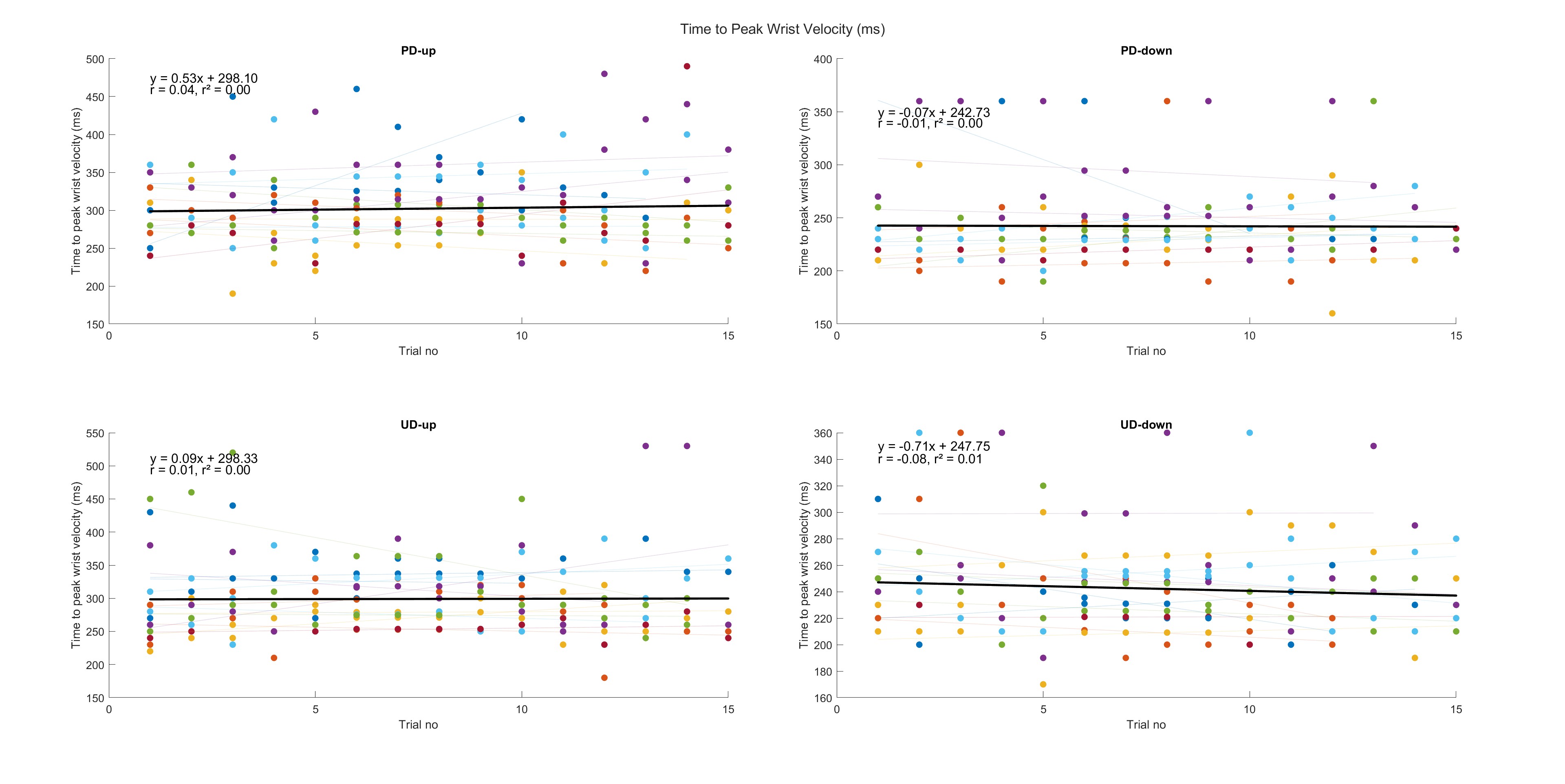


**Figure S3.** Regression analysis of wrist kinematics across trials. Time to peak wrist velocity as a function of trial number, with individual data points similarly color-coded per participant. The bold black line shows the group-level regression for this variable.

**Table S2.** The results of t-test for dependent samples for kinematic variables. Table shows the differences between control condition and perturbed conditions.

| Variable |  | Control vs. | | | |
| --- | --- | --- | --- | --- | --- |
|  |  | UD-up | PD-up | UD-down | PD-down |
| Peak aperture velocity |  |  |  |  |  |
|  | t | -0.99 | -0.94 | -1.03 | -1.15 |
|  | *p* | 0.343 | 0.363 | 0.321 | 0.273 |
| Time to peak aperture velocity |  |  |  |  |  |
|  | t | 1.93 | 1.24 | 3.55 | 3.47 |
|  | *p* | 0.077 | 0.237 | **0.004** | **0.005** |
| Aperture acceleration |  |  |  |  |  |
|  | t | -2.68 | -1.55 | -1.70 | -1.84 |
|  | *p* | 0.020 | 0.147 | 0.116 | 0.091 |
| Time to peak aperture acceleration |  |  |  |  |  |
|  | t | 4.76 | 4.40 | 3.85 | 3.55 |
|  | *p* | **<0.001** | **0.001** | **0.002** | **0.004** |
| Wrist acceleration |  |  |  |  |  |
|  | t | -2.68 | -2.43 | -4.22 | -3.43 |
|  | *p* | 0.020 | 0.032 | **0.001** | **0.005** |
| Time to peak wrist acceleration |  |  |  |  |  |
|  | t | 3.39 | 3.46 | 4.80 | 5.59 |
|  | *p* | **0.005** | **0.005** | **<0.001** | **<0.001** |

**Table S3.** The results of Wilcoxon signed-rank comparison with Bonferroni correction for iEMG in 50 ms time frames. Table shows the differences between control condition and perturbed conditions.

| **Msl** | **Condition** | **MP1** | | **MP 2** | | **MP 3** | | **MP 4** | | **APA1** | | **APA2** | | **APA3** | | **APA4** | | **CPA1** | | **CPA2** | | **CPA3** | | **CPA4** | |
| --- | --- | --- | --- | --- | --- | --- | --- | --- | --- | --- | --- | --- | --- | --- | --- | --- | --- | --- | --- | --- | --- | --- | --- | --- | --- |
|  | **Control vs.** | Z | p | Z | p | Z | p | Z | p | Z | p | Z | p | Z | p | Z | p | Z | p | Z | p | Z | p | Z | p |
| **FDI** | UD-up | 0.52 | 0.600 | 1.85 | 0.064 | 0.59 | 0.552 | 0.94 | 0.345 | 1.57 | 0.116 | 1.15 | 0.249 | 1.15 | 0.249 | 2.34 | 0.019 | 2.90 | **0.004** | 2.83 | **0.005** | 1.29 | 0.196 | 0.52 | 0.600 |
|  | PD-up | 0.24 | 0.807 | 0.24 | 0.807 | 0.94 | 0.345 | 1.01 | 0.311 | 0.03 | 0.972 | 0.17 | 0.861 | 1.99 | 0.046 | 2.13 | 0.033 | 3.11 | **0.002** | 3.18 | **0.001** | 1.43 | 0.152 | 0.10 | 0.917 |
|  | UD-down | 0.17 | 0.861 | 0.24 | 0.807 | 0.17 | 0.861 | 0.03 | 0.972 | 0.10 | 0.917 | 0.17 | 0.861 | 0.24 | 0.807 | 0.80 | 0.422 | 1.57 | 0.116 | 2.27 | 0.023 | 1.71 | 0.087 | 1.43 | 0.152 |
|  | PD-down | 0.87 | 0.382 | 1.71 | 0.087 | 0.87 | 0.382 | 1.57 | 0.116 | 1.92 | 0.055 | 2.41 | 0.016 | 1.78 | 0.075 | 1.43 | 0.152 | 2.76 | **0.006** | 3.11 | **0.002** | 2.83 | **0.005** | 2.06 | 0.039 |
| **EI** | UD-up | 1.01 | 0.311 | 1.92 | 0.055 | 2.06 | 0.039 | 2.27 | 0.023 | 1.92 | 0.055 | 1.29 | 0.196 | 1.29 | 0.196 | 2.48 | 0.013 | 2.06 | 0.039 | 1.64 | 0.101 | 1.36 | 0.173 | 1.08 | 0.279 |
|  | PD-up | 1.01 | 0.311 | 1.57 | 0.116 | 1.50 | 0.133 | 0.03 | 0.972 | 1.22 | 0.221 | 1.50 | 0.133 | 1.64 | 0.101 | 2.20 | 0.028 | 1.64 | 0.101 | 1.29 | 0.196 | 0.45 | 0.650 | 0.80 | 0.422 |
|  | UD-down | 1.22 | 0.221 | 1.29 | 0.196 | 2.41 | 0.016 | 1.78 | 0.075 | 0.94 | 0.345 | 1.36 | 0.173 | 1.43 | 0.152 | 2.27 | 0.023 | 2.76 | **0.006** | 2.55 | **0.011** | 1.50 | 0.133 | 0.59 | 0.552 |
|  | PD-down | 1.36 | 0.173 | 1.78 | 0.075 | 2.48 | 0.013 | 2.83 | **0.005** | 2.06 | 0.039 | 2.13 | 0.033 | 1.57 | 0.116 | 2.20 | 0.028 | 2.69 | **0.007** | 2.41 | 0.016 | 1.29 | 0.196 | 1.08 | 0.279 |
| **APB** | UD-up | 1.15 | 0.249 | 0.38 | 0.701 | 1.15 | 0.249 | 0.59 | 0.552 | 1.64 | 0.101 | 1.01 | 0.311 | 1.08 | 0.279 | 1.57 | 0.116 | 1.08 | 0.279 | 0.94 | 0.345 | 1.57 | 0.116 | 1.01 | 0.311 |
|  | PD-up | 0.87 | 0.382 | 0.52 | 0.600 | 1.22 | 0.221 | 0.03 | 0.972 | 1.01 | 0.311 | 1.57 | 0.116 | 2.27 | 0.023 | 1.99 | 0.046 | 1.22 | 0.221 | 0.80 | 0.422 | 1.29 | 0.196 | 0.03 | 0.972 |
|  | UD-down | 1.08 | 0.279 | 0.66 | 0.507 | 0.17 | 0.861 | 0.03 | 0.972 | 0.17 | 0.861 | 1.71 | 0.087 | 2.34 | 0.019 | 2.20 | 0.028 | 2.48 | 0.013 | 2.69 | **0.007** | 2.97 | **0.003** | 1.85 | 0.064 |
|  | PD-down | 0.52 | 0.600 | 0.03 | 0.972 | 1.85 | 0.064 | 0.38 | 0.701 | 0.45 | 0.650 | 1.15 | 0.249 | 1.29 | 0.196 | 1.43 | 0.152 | 1.78 | 0.075 | 2.69 | **0.007** | 2.76 | **0.006** | 2.06 | 0.039 |
| **EPB** | UD-up | 2.41 | 0.016 | 2.55 | **0.011** | 1.85 | 0.064 | 1.64 | 0.101 | 1.01 | 0.311 | 1.29 | 0.196 | 1.57 | 0.116 | 2.41 | 0.016 | 1.78 | 0.075 | 0.59 | 0.552 | 0.24 | 0.807 | 0.03 | 0.972 |
|  | PD-up | 0.87 | 0.382 | 0.87 | 0.382 | 1.99 | 0.046 | 0.94 | 0.345 | 2.06 | 0.039 | 1.92 | 0.055 | 1.92 | 0.055 | 1.92 | 0.055 | 1.78 | 0.075 | 0.38 | 0.701 | 0.10 | 0.917 | 0.17 | 0.861 |
|  | UD-down | 0.38 | 0.701 | 1.43 | 0.152 | 1.92 | 0.055 | 1.71 | 0.087 | 1.50 | 0.133 | 1.99 | 0.046 | 2.27 | 0.023 | 1.99 | 0.046 | 2.97 | **0.003** | 2.76 | **0.006** | 2.06 | 0.039 | 1.01 | 0.311 |
|  | PD-down | 0.94 | 0.345 | 0.80 | 0.422 | 1.43 | 0.152 | 1.64 | 0.101 | 1.29 | 0.196 | 1.08 | 0.279 | 2.06 | 0.039 | 2.34 | 0.019 | 2.55 | **0.011** | 2.62 | **0.009** | 1.85 | 0.064 | 0.73 | 0.463 |
| **FDS** | UD-up | 0.52 | 0.600 | 0.03 | 0.972 | 0.87 | 0.382 | 1.92 | 0.055 | 2.90 | **0.004** | 1.99 | 0.046 | 2.76 | **0.006** | 3.04 | **0.002** | 3.18 | **0.001** | 3.18 | **0.001** | 3.18 | **0.001** | 3.11 | **0.002** |
|  | PD-up | 0.66 | 0.507 | 1.99 | 0.046 | 2.27 | 0.023 | 1.71 | 0.087 | 1.78 | 0.075 | 2.62 | **0.009** | 2.62 | **0.009** | 2.90 | **0.004** | 2.97 | **0.003** | 3.18 | **0.001** | 3.18 | **0.001** | 2.90 | **0.004** |
|  | UD-down | 0.66 | 0.507 | 1.36 | 0.173 | 0.24 | 0.807 | 0.66 | 0.507 | 0.38 | 0.701 | 1.22 | 0.221 | 1.15 | 0.249 | 1.29 | 0.196 | 2.97 | **0.003** | 2.27 | 0.023 | 1.08 | 0.279 | 1.85 | 0.064 |
|  | PD-down | 0.31 | 0.753 | 0.66 | 0.507 | 1.01 | 0.311 | 1.64 | 0.101 | 1.78 | 0.075 | 1.78 | 0.075 | 2.20 | 0.028 | 2.69 | **0.007** | 3.18 | **0.001** | 2.55 | **0.011** | 2.34 | 0.019 | 2.34 | 0.019 |
| **EDC** | UD-up | 1.01 | 0.311 | 1.36 | 0.173 | 1.78 | 0.075 | 1.99 | 0.046 | 2.13 | 0.033 | 1.43 | 0.152 | 1.99 | 0.046 | 3.11 | **0.002** | 2.83 | **0.005** | 0.10 | 0.917 | 0.73 | 0.463 | 1.22 | 0.221 |
|  | PD-up | 0.94 | 0.345 | 1.57 | 0.116 | 2.62 | **0.009** | 1.01 | 0.311 | 1.78 | 0.075 | 2.48 | 0.013 | 2.69 | **0.007** | 3.18 | **0.001** | 3.18 | **0.001** | 0.31 | 0.753 | 1.01 | 0.311 | 1.43 | 0.152 |
|  | UD-down | 1.85 | 0.064 | 1.29 | 0.196 | 1.85 | 0.064 | 0.59 | 0.552 | 1.22 | 0.221 | 1.57 | 0.116 | 1.01 | 0.311 | 2.55 | **0.011** | 3.18 | **0.001** | 3.18 | **0.001** | 2.76 | **0.006** | 1.99 | 0.046 |
|  | PD-down | 0.31 | 0.753 | 0.73 | 0.463 | 1.15 | 0.249 | 2.62 | **0.009** | 2.41 | 0.016 | 1.85 | 0.064 | 2.90 | **0.004** | 3.18 | **0.001** | 3.18 | **0.001** | 3.18 | **0.001** | 3.04 | **0.002** | 2.69 | **0.007** |
| **AD** | UD-up | 0.10 | 0.917 | 1.64 | 0.101 | 1.08 | 0.279 | 0.24 | 0.807 | 1.01 | 0.311 | 0.80 | 0.422 | 0.45 | 0.650 | 0.94 | 0.345 | 1.78 | 0.075 | 0.38 | 0.701 | 0.59 | 0.552 | 0.80 | 0.422 |
|  | PD-up | 0.03 | 0.972 | 0.94 | 0.345 | 1.22 | 0.221 | 0.52 | 0.600 | 0.38 | 0.701 | 0.52 | 0.600 | 1.36 | 0.173 | 1.78 | 0.075 | 2.27 | 0.023 | 0.66 | 0.507 | 1.85 | 0.064 | 2.41 | 0.016 |
|  | UD-down | 0.24 | 0.807 | 0.94 | 0.345 | 0.73 | 0.463 | 1.85 | 0.064 | 0.17 | 0.861 | 1.08 | 0.279 | 0.59 | 0.552 | 0.03 | 0.972 | 1.57 | 0.116 | 2.48 | 0.013 | 3.11 | **0.002** | 3.11 | **0.002** |
|  | PD-down | 0.03 | 0.972 | 0.03 | 0.972 | 0.66 | 0.507 | 0.24 | 0.807 | 0.10 | 0.917 | 0.59 | 0.552 | 1.22 | 0.221 | 0.87 | 0.382 | 1.64 | 0.101 | 3.18 | **0.001** | 3.18 | **0.001** | 2.69 | **0.007** |
| **PD** | UD-up | 3.04 | **0.002** | 2.76 | **0.006** | 1.85 | 0.064 | 0.94 | 0.345 | 0.17 | 0.861 | 0.24 | 0.807 | 0.59 | 0.552 | 1.64 | 0.101 | 0.73 | 0.463 | 0.10 | 0.917 | 0.17 | 0.861 | 0.52 | 0.600 |
|  | PD-up | 2.20 | 0.028 | 2.41 | 0.016 | 0.24 | 0.807 | 0.03 | 0.972 | 1.22 | 0.221 | 0.66 | 0.507 | 0.73 | 0.463 | 1.50 | 0.133 | 1.78 | 0.075 | 0.38 | 0.701 | 0.59 | 0.552 | 0.73 | 0.463 |
|  | UD-down | 1.64 | 0.101 | 1.92 | 0.055 | 0.52 | 0.600 | 0.94 | 0.345 | 0.10 | 0.917 | 0.17 | 0.861 | 0.94 | 0.345 | 1.08 | 0.279 | 3.04 | **0.002** | 2.34 | 0.019 | 1.57 | 0.116 | 1.08 | 0.279 |
|  | PD-down | 1.22 | 0.221 | 1.50 | 0.133 | 1.64 | 0.101 | 0.59 | 0.552 | 0.17 | 0.861 | 0.73 | 0.463 | 1.92 | 0.055 | 1.85 | 0.064 | 2.62 | **0.009** | 2.76 | **0.006** | 1.15 | 0.249 | 1.36 | 0.173 |
| **BB** | UD-up | 0.38 | 0.701 | 0.24 | 0.807 | 1.22 | 0.221 | 1.78 | 0.075 | 1.43 | 0.152 | 2.27 | 0.023 | 1.71 | 0.087 | 1.57 | 0.116 | 0.73 | 0.463 | 0.03 | 0.972 | 0.73 | 0.463 | 1.29 | 0.196 |
|  | PD-up | 0.17 | 0.861 | 0.73 | 0.463 | 0.66 | 0.507 | 1.15 | 0.249 | 0.59 | 0.552 | 0.94 | 0.345 | 1.01 | 0.311 | 2.13 | 0.033 | 0.94 | 0.345 | 0.10 | 0.917 | 0.94 | 0.345 | 1.22 | 0.221 |
|  | UD-down | 0.45 | 0.650 | 1.36 | 0.173 | 0.17 | 0.861 | 1.29 | 0.196 | 0.03 | 0.972 | 1.01 | 0.311 | 1.15 | 0.249 | 1.92 | 0.055 | 3.11 | **0.002** | 3.18 | **0.001** | 3.18 | **0.001** | 3.18 | **0.001** |
|  | PD-down | 0.03 | 0.972 | 0.10 | 0.917 | 1.92 | 0.055 | 1.78 | 0.075 | 0.66 | 0.507 | 0.94 | 0.345 | 0.87 | 0.382 | 1.99 | 0.046 | 3.18 | **0.001** | 3.18 | **0.001** | 3.18 | **0.001** | 3.18 | **0.001** |
| **TB** | UD-up | 0.03 | 0.972 | 0.10 | 0.917 | 0.03 | 0.972 | 0.87 | 0.382 | 0.94 | 0.345 | 0.66 | 0.507 | 1.57 | 0.116 | 2.83 | **0.005** | 3.11 | **0.002** | 3.18 | **0.001** | 2.76 | **0.006** | 2.83 | **0.005** |
|  | PD-up | 1.43 | 0.152 | 0.24 | 0.807 | 0.94 | 0.345 | 1.15 | 0.249 | 1.36 | 0.173 | 2.34 | 0.019 | 1.57 | 0.116 | 2.13 | 0.033 | 3.18 | **0.001** | 3.11 | **0.002** | 3.18 | **0.001** | 3.04 | **0.002** |
|  | UD-down | 1.08 | 0.279 | 0.87 | 0.382 | 1.36 | 0.173 | 2.27 | 0.023 | 1.57 | 0.116 | 1.85 | 0.064 | 1.50 | 0.133 | 2.97 | **0.003** | 3.18 | **0.001** | 3.18 | **0.001** | 3.18 | **0.001** | 3.18 | **0.001** |
|  | PD-down | 0.03 | 0.972 | 0.10 | 0.917 | 0.59 | 0.552 | 0.45 | 0.650 | 1.64 | 0.101 | 2.41 | 0.016 | 2.27 | 0.023 | 2.48 | 0.013 | 3.18 | **0.001** | 3.18 | **0.001** | 3.18 | **0.001** | 3.18 | **0.001** |
| **Esr** | UD-up | 0.87 | 0.382 | 0.94 | 0.345 | 2.06 | 0.039 | 0.24 | 0.807 | 0.31 | 0.753 | 0.66 | 0.507 | 1.64 | 0.101 | 0.03 | 0.972 | 1.64 | 0.101 | 3.18 | **0.001** | 3.18 | **0.001** | 2.76 | **0.006** |
|  | PD-up | 0.80 | 0.422 | 0.52 | 0.600 | 0.45 | 0.650 | 0.03 | 0.972 | 0.59 | 0.552 | 1.78 | 0.075 | 1.99 | 0.046 | 1.92 | 0.055 | 2.55 | **0.011** | 3.18 | **0.001** | 3.18 | **0.001** | 2.97 | **0.003** |
|  | UD-down | 2.48 | 0.013 | 1.50 | 0.133 | 0.94 | 0.345 | 1.43 | 0.152 | 2.06 | 0.039 | 1.15 | 0.249 | 1.99 | 0.046 | 1.43 | 0.152 | 1.29 | 0.196 | 3.11 | **0.002** | 2.83 | **0.005** | 2.20 | 0.028 |
|  | PD-down | 1.92 | 0.055 | 1.57 | 0.116 | 1.57 | 0.116 | 1.78 | 0.075 | 1.22 | 0.221 | 1.64 | 0.101 | 1.29 | 0.196 | 0.66 | 0.507 | 0.59 | 0.552 | 3.11 | **0.002** | 2.76 | **0.006** | 2.13 | 0.033 |
| **Esl** | UD-up | 0.03 | 0.972 | 0.45 | 0.650 | 0.38 | 0.701 | 1.15 | 0.249 | 0.31 | 0.753 | 1.01 | 0.311 | 0.10 | 0.917 | 1.29 | 0.196 | 0.94 | 0.345 | 2.48 | 0.013 | 2.97 | **0.003** | 2.83 | **0.005** |
|  | PD-up | 1.71 | 0.087 | 0.94 | 0.345 | 0.52 | 0.600 | 1.08 | 0.279 | 0.10 | 0.917 | 1.01 | 0.311 | 0.59 | 0.552 | 0.87 | 0.382 | 1.50 | 0.133 | 2.90 | **0.004** | 3.11 | **0.002** | 2.69 | **0.007** |
|  | UD-down | 2.20 | 0.028 | 0.17 | 0.861 | 0.38 | 0.701 | 0.31 | 0.753 | 0.10 | 0.917 | 1.29 | 0.196 | 0.17 | 0.861 | 0.10 | 0.917 | 2.34 | 0.019 | 3.18 | **0.001** | 3.18 | **0.001** | 3.18 | **0.001** |
|  | PD-down | 0.45 | 0.650 | 0.87 | 0.382 | 0.17 | 0.861 | 0.38 | 0.701 | 0.45 | 0.650 | 1.22 | 0.221 | 2.27 | 0.023 | 1.22 | 0.221 | 2.20 | 0.028 | 3.18 | **0.001** | 3.11 | **0.002** | 3.18 | **0.001** |
| **PEC** | UD-up | 1.85 | 0.064 | 1.36 | 0.173 | 1.50 | 0.133 | 2.27 | 0.023 | 1.57 | 0.116 | 2.34 | 0.019 | 2.27 | 0.023 | 2.83 | **0.005** | 3.18 | **0.001** | 3.18 | **0.001** | 3.18 | **0.001** | 3.18 | **0.001** |
|  | PD-up | 1.92 | 0.055 | 2.20 | 0.028 | 0.59 | 0.552 | 0.10 | 0.917 | 1.43 | 0.152 | 1.29 | 0.196 | 1.71 | 0.087 | 2.34 | 0.019 | 3.11 | **0.002** | 3.18 | **0.001** | 3.18 | **0.001** | 3.18 | **0.001** |
|  | UD-down | 1.08 | 0.279 | 0.52 | 0.600 | 0.80 | 0.422 | 0.66 | 0.507 | 0.31 | 0.753 | 1.99 | 0.046 | 1.64 | 0.101 | 2.27 | 0.023 | 3.18 | **0.001** | 3.18 | **0.001** | 3.18 | **0.001** | 3.18 | **0.001** |
|  | PD-down | 0.80 | 0.422 | 0.87 | 0.382 | 1.92 | 0.055 | 2.62 | **0.009** | 2.76 | **0.006** | 2.76 | **0.006** | 2.41 | 0.016 | 2.41 | 0.016 | 3.18 | **0.001** | 3.18 | **0.001** | 3.18 | **0.001** | 3.18 | **0.001** |
| **UT** | UD-up | 0.80 | 0.422 | 0.03 | 0.972 | 0.10 | 0.917 | 0.45 | 0.650 | 0.59 | 0.552 | 0.59 | 0.552 | 1.15 | 0.249 | 1.08 | 0.279 | 2.48 | 0.013 | 0.66 | 0.507 | 2.76 | **0.006** | 1.36 | 0.173 |
|  | PD-up | 0.73 | 0.463 | 1.15 | 0.249 | 0.59 | 0.552 | 1.08 | 0.279 | 0.45 | 0.650 | 1.64 | 0.101 | 0.03 | 0.972 | 0.03 | 0.972 | 2.76 | **0.006** | 0.24 | 0.807 | 1.85 | 0.064 | 0.52 | 0.600 |
|  | UD-down | 1.22 | 0.221 | 1.36 | 0.173 | 0.80 | 0.422 | 0.59 | 0.552 | 0.38 | 0.701 | 1.29 | 0.196 | 1.29 | 0.196 | 1.36 | 0.173 | 3.04 | **0.002** | 2.48 | 0.013 | 2.69 | **0.007** | 3.04 | **0.002** |
|  | PD-down | 1.36 | 0.173 | 0.52 | 0.600 | 0.73 | 0.463 | 0.73 | 0.463 | 0.73 | 0.463 | 0.59 | 0.552 | 0.87 | 0.382 | 0.66 | 0.507 | 2.90 | **0.004** | 1.78 | 0.075 | 1.99 | 0.046 | 3.11 | **0.002** |
| **LAT** | UD-up | 0.80 | 0.422 | 0.80 | 0.422 | 0.17 | 0.861 | 1.22 | 0.221 | 0.45 | 0.650 | 0.17 | 0.861 | 0.38 | 0.701 | 0.45 | 0.650 | 1.08 | 0.279 | 1.50 | 0.133 | 2.27 | 0.023 | 2.76 | **0.006** |
|  | PD-up | 1.15 | 0.249 | 0.87 | 0.382 | 0.80 | 0.422 | 0.31 | 0.753 | 0.24 | 0.807 | 0.66 | 0.507 | 0.10 | 0.917 | 0.59 | 0.552 | 0.03 | 0.972 | 1.57 | 0.116 | 2.48 | 0.013 | 2.34 | 0.019 |
|  | UD-down | 0.80 | 0.422 | 1.92 | 0.055 | 0.80 | 0.422 | 0.73 | 0.463 | 0.38 | 0.701 | 1.29 | 0.196 | 0.17 | 0.861 | 0.59 | 0.552 | 2.90 | **0.004** | 3.18 | **0.001** | 3.11 | **0.002** | 3.04 | **0.002** |
|  | PD-down | 1.08 | 0.279 | 1.15 | 0.249 | 0.59 | 0.552 | 0.10 | 0.917 | 0.17 | 0.861 | 0.45 | 0.650 | 0.73 | 0.463 | 1.29 | 0.196 | 2.90 | **0.004** | 3.11 | **0.002** | 3.18 | **0.001** | 3.04 | **0.002** |
| **SER** | UD-up | 0.03 | 0.972 | 0.66 | 0.507 | 1.08 | 0.279 | 1.78 | 0.075 | 1.71 | 0.087 | 0.10 | 0.917 | 0.87 | 0.382 | 0.59 | 0.552 | 1.92 | 0.055 | 2.27 | 0.023 | 2.90 | **0.004** | 2.83 | **0.005** |
|  | PD-up | 0.24 | 0.807 | 0.66 | 0.507 | 0.10 | 0.917 | 1.64 | 0.101 | 2.27 | 0.023 | 0.87 | 0.382 | 1.57 | 0.116 | 2.06 | 0.039 | 1.57 | 0.116 | 2.55 | **0.011** | 3.11 | **0.002** | 3.11 | **0.002** |
|  | UD-down | 1.22 | 0.221 | 0.45 | 0.650 | 0.87 | 0.382 | 1.01 | 0.311 | 1.22 | 0.221 | 0.10 | 0.917 | 1.36 | 0.173 | 1.71 | 0.087 | 3.18 | **0.001** | 3.18 | **0.001** | 3.18 | **0.001** | 3.18 | **0.001** |
|  | PD-down | 0.94 | 0.345 | 0.17 | 0.861 | 1.29 | 0.196 | 1.43 | 0.152 | 0.03 | 0.972 | 0.45 | 0.650 | 0.17 | 0.861 | 2.34 | 0.019 | 3.18 | **0.001** | 3.18 | **0.001** | 3.18 | **0.001** | 3.18 | **0.001** |

**Table S4**. The results of 2 × 2 repeated measures ANOVA for kinematic variables.

| Variable | Main effect | F | p |
| --- | --- | --- | --- |
| Peak aperture velocity |  |  |  |
|  | DIR | 0.07 | 0.794 |
|  | INS | 0.16 | 0.697 |
|  | DIR × INS | 0.04 | 0.840 |
| Time to peak aperture velocity |  |  |  |
|  | DIR | 11.71 | **0.005** |
|  | INS | 3.00 | 0.109 |
|  | DIR × INS | 0.56 | 0.467 |
| Aperture acceleration |  |  |  |
|  | DIR | 0.59 | 0.457 |
|  | INS | 0.37 | 0.553 |
|  | DIR × INS | 1.03 | 0.329 |
| Time to peak aperture acceleration |  |  |  |
|  | DIR | 0.47 | 0.508 |
|  | INS | 1.10 | 0.315 |
|  | DIR × INS | 0.07 | 0.800 |
| Wrist acceleration |  |  |  |
|  | DIR | 0.29 | 0.598 |
|  | INS | 0.00 | 0.981 |
|  | DIR × INS | 2.66 | 0.129 |
| Time to peak wrist acceleration |  |  |  |
|  | DIR | 7.68 | **0.017** |
|  | INS | 0.08 | 0.780 |
|  | DIR × INS | 0.09 | 0.771 |

Legend: DIR – Direction, INS – Instruction, DIR × INS – interaction between Direction and Instruction

**Table S5.** The results of 2 × 2 repeated measures ANOVA for iEMG in 50 ms time frames.

| **Msl** | **Main effect** | **MP1** | | **MP 2** | | **MP 3** | | **MP 4** | | **APA1** | | **APA2** | | **APA3** | | **APA4** | | **CPA1** | | **CPA2** | | **CPA3** | | **CPA4** | |
| --- | --- | --- | --- | --- | --- | --- | --- | --- | --- | --- | --- | --- | --- | --- | --- | --- | --- | --- | --- | --- | --- | --- | --- | --- | --- |
|  |  | F | p | F | p | F | p | F | p | F | p | F | p | F | p | F | p | F | p | F | p | F | p | F | p |
| **FDI** | DIR | 2.42 | 0.146 | 1.66 | 0.221 | 0.16 | 1.174 | 0.63 | 0.777 | 4.39 | 1.099 | 0.12 | 0.195 | 24.37 | 0.886 | 125.92 | 0.752 | 149.71 | 0.584 | 30.85 | 0.956 | 54.70 | 1.538 | 64.92 | 1.410 |
|  | INS | 0.09 | 0.771 | 2.41 | 0.146 | 0.64 | 1.559 | 2.07 | 0.728 | 5.40 | 0.921 | 0.22 | 0.304 | 52.65 | 0.955 | 499.25 | 1.010 | 336.12 | 1.016 | 89.61 | 1.184 | 63.10 | 1.031 | 26.10 | 0.962 |
|  | DIR × INS | 1.34 | 0.269 | 0.98 | 0.341 | 1.62 | 1.216 | 24.20 | 1.148 | 65.81 | 1.236 | 18.80 | 1.824 | 32.53 | 1.175 | 213.03 | 1.018 | 220.02 | 0.933 | 123.57 | 0.975 | 0.98 | 0.605 | 0.25 | 0.563 |
| **EI** | DIR | 0.79 | 0.393 | 2.84 | 0.118 | 1.05 | 0.325 | 2.77 | 0.122 | 0.87 | 0.370 | 0.40 | 0.538 | 0.75 | 0.402 | 1.23 | 0.290 | 0.20 | 0.661 | 0.47 | 0.504 | 0.12 | 0.740 | 0.15 | 0.709 |
|  | INS | 0.58 | 0.462 | 0.02 | 0.903 | 1.02 | 0.332 | 1.88 | 0.195 | 0.30 | 0.593 | 0.41 | 0.536 | 0.70 | 0.419 | 3.41 | 0.090 | 2.77 | 0.122 | 0.04 | 0.837 | 1.67 | 0.221 | 3.77 | 0.076 |
|  | DIR × INS | 0.27 | 0.616 | 1.32 | 0.273 | 4.31 | 0.060 | 6.22 | **0.028** | 3.10 | 0.104 | 0.12 | 0.730 | 0.03 | 0.860 | 0.78 | 0.393 | 0.74 | 0.407 | 0.00 | 0.952 | 2.02 | 0.181 | 1.78 | 0.207 |
| **APB** | DIR | 0.08 | 0.776 | 0.13 | 0.721 | 0.94 | 0.351 | 0.71 | 0.416 | 1.76 | 0.209 | 0.02 | 0.887 | 1.72 | 0.214 | 0.36 | 0.558 | 2.94 | 0.112 | 2.61 | 0.132 | 0.98 | 0.341 | 1.89 | 0.194 |
|  | INS | 0.08 | 0.780 | 0.63 | 0.442 | 2.41 | 0.147 | 0.90 | 0.362 | 0.32 | 0.585 | 0.49 | 0.499 | 0.41 | 0.535 | 0.02 | 0.892 | 0.18 | 0.682 | 0.02 | 0.893 | 0.58 | 0.462 | 0.03 | 0.871 |
|  | DIR × INS | 0.47 | 0.505 | 0.76 | 0.400 | 0.89 | 0.364 | 0.41 | 0.536 | 0.03 | 0.871 | 0.97 | 0.344 | 0.11 | 0.751 | 0.33 | 0.575 | 0.19 | 0.673 | 1.85 | 0.199 | 1.62 | 0.227 | 1.46 | 0.250 |
| **EPB** | DIR | 0.33 | 0.575 | 0.02 | 0.889 | 0.00 | 0.985 | 0.09 | 0.771 | 0.86 | 0.371 | 0.60 | 0.452 | 0.00 | 0.961 | 0.18 | 0.677 | 12.33 | **0.004** | 7.90 | **0.016** | 7.06 | **0.021** | 5.00 | **0.045** |
|  | INS | 0.33 | 0.576 | 0.05 | 0.822 | 1.45 | 0.252 | 0.46 | 0.512 | 0.31 | 0.587 | 0.48 | 0.501 | 1.09 | 0.316 | 1.23 | 0.290 | 1.09 | 0.317 | 2.28 | 0.157 | 1.83 | 0.201 | 3.19 | 0.099 |
|  | DIR × INS | 4.32 | 0.060 | 1.56 | 0.236 | 0.04 | 0.845 | 0.45 | 0.513 | 0.00 | 0.972 | 0.08 | 0.781 | 0.43 | 0.524 | 3.14 | 0.102 | 0.78 | 0.394 | 0.17 | 0.691 | 0.20 | 0.662 | 0.08 | 0.779 |
| **FDS** | DIR | 1.79 | 0.206 | 2.71 | 0.126 | 0.96 | 0.347 | 1.57 | 0.235 | 3.01 | 0.108 | 1.57 | 0.235 | 2.32 | 0.154 | 5.53 | **0.037** | 1.70 | 0.217 | 5.06 | **0.044** | 5.34 | **0.039** | 1.37 | 0.264 |
|  | INS | 4.12 | 0.065 | 4.33 | 0.060 | 2.44 | 0.144 | 0.24 | 0.631 | 0.19 | 0.667 | 0.01 | 0.932 | 2.11 | 0.172 | 1.16 | 0.303 | 0.87 | 0.370 | 0.29 | 0.602 | 0.23 | 0.642 | 0.00 | 0.989 |
|  | DIR × INS | 0.69 | 0.421 | 0.00 | 0.987 | 0.31 | 0.587 | 0.86 | 0.373 | 4.05 | 0.067 | 1.05 | 0.325 | 2.76 | 0.122 | 1.91 | 0.193 | 2.05 | 0.178 | 4.56 | 0.054 | 1.65 | 0.224 | 1.77 | 0.208 |
| **EDC** | DIR | 1.19 | 0.296 | 0.92 | 0.355 | 0.97 | 0.344 | 0.02 | 0.878 | 1.42 | 0.257 | 0.33 | 0.578 | 0.55 | 0.473 | 1.40 | 0.260 | 0.02 | 0.888 | 3.61 | 0.082 | 15.67 | **0.002** | 22.84 | **<0.001** |
|  | INS | 0.29 | 0.601 | 1.30 | 0.277 | 0.22 | 0.644 | 1.12 | 0.311 | 0.92 | 0.357 | 0.74 | 0.408 | 1.29 | 0.278 | 1.74 | 0.212 | 2.04 | 0.178 | 1.51 | 0.243 | 0.99 | 0.340 | 1.80 | 0.205 |
|  | DIR × INS | 0.07 | 0.789 | 0.27 | 0.610 | 1.11 | 0.312 | 5.08 | **0.044** | 1.51 | 0.243 | 0.50 | 0.493 | 1.33 | 0.270 | 1.64 | 0.225 | 1.83 | 0.201 | 3.38 | 0.091 | 1.51 | 0.243 | 8.10 | **0.015** |
| **AD** | DIR | 0.49 | 0.499 | 1.77 | 0.208 | 0.52 | 0.484 | 3.60 | 0.082 | 0.72 | 0.414 | 0.16 | 0.699 | 0.26 | 0.617 | 0.09 | 0.770 | 17.89 | **0.001** | 41.16 | **<0.001** | 57.20 | **<0.001** | 23.43 | **<0.001** |
|  | INS | 0.00 | 0.963 | 0.59 | 0.458 | 0.02 | 0.896 | 0.39 | 0.544 | 0.58 | 0.463 | 1.49 | 0.246 | 0.00 | 0.963 | 0.62 | 0.448 | 0.16 | 0.698 | 0.63 | 0.443 | 0.52 | 0.484 | 0.01 | 0.922 |
|  | DIR × INS | 0.30 | 0.593 | 1.18 | 0.298 | 0.00 | 0.971 | 0.01 | 0.919 | 0.55 | 0.472 | 2.22 | 0.162 | 2.04 | 0.179 | 0.81 | 0.385 | 0.10 | 0.759 | 0.19 | 0.671 | 16.58 | **0.002** | 6.93 | **0.022** |
| **PD** | DIR | 1.86 | 0.198 | 1.85 | 0.199 | 0.02 | 0.899 | 0.13 | 0.724 | 0.13 | 0.723 | 3.47 | 0.087 | 3.66 | 0.080 | 2.51 | 0.139 | 7.74 | **0.017** | 12.95 | **0.004** | 3.36 | 0.092 | 0.01 | 0.932 |
|  | INS | 1.22 | 0.291 | 0.52 | 0.485 | 0.95 | 0.349 | 3.99 | 0.069 | 1.99 | 0.184 | 3.40 | 0.090 | 4.82 | **0.049** | 2.49 | 0.140 | 3.68 | 0.079 | 0.61 | 0.451 | 2.57 | 0.135 | 0.55 | 0.471 |
|  | DIR × INS | 0.74 | 0.406 | 0.49 | 0.497 | 2.63 | 0.131 | 0.04 | 0.848 | 0.06 | 0.803 | 1.09 | 0.316 | 1.34 | 0.270 | 1.56 | 0.235 | 2.01 | 0.182 | 0.46 | 0.509 | 0.01 | 0.940 | 1.07 | 0.321 |
| **BB** | DIR | 0.93 | 0.354 | 0.19 | 0.674 | 0.18 | 0.682 | 0.02 | 0.900 | 0.05 | 0.835 | 2.44 | 0.144 | 0.42 | 0.529 | 0.26 | 0.616 | 16.29 | **0.002** | 18.37 | **0.001** | 10.93 | **0.006** | 8.74 | **0.012** |
|  | INS | 0.78 | 0.395 | 0.90 | 0.361 | 0.07 | 0.792 | 0.25 | 0.625 | 2.67 | 0.128 | 6.64 | **0.024** | 4.67 | 0.052 | 0.07 | 0.798 | 4.54 | 0.054 | 0.04 | 0.840 | 0.22 | 0.645 | 1.72 | 0.214 |
|  | DIR × INS | 0.04 | 0.838 | 0.00 | 0.969 | 1.96 | 0.187 | 0.15 | 0.708 | 0.19 | 0.671 | 1.86 | 0.198 | 0.29 | 0.600 | 0.14 | 0.715 | 1.20 | 0.294 | 0.21 | 0.652 | 0.01 | 0.909 | 0.90 | 0.363 |
| **TB** | DIR | 0.95 | 0.350 | 0.40 | 0.537 | 0.15 | 0.705 | 0.46 | 0.510 | 0.78 | 0.395 | 1.73 | 0.213 | 0.49 | 0.498 | 0.58 | 0.461 | 2.63 | 0.131 | 6.89 | **0.022** | 7.52 | **0.018** | 6.96 | **0.022** |
|  | INS | 0.50 | 0.494 | 1.39 | 0.262 | 0.79 | 0.391 | 0.45 | 0.516 | 1.24 | 0.288 | 2.03 | 0.180 | 2.47 | 0.142 | 0.05 | 0.821 | 1.39 | 0.261 | 1.00 | 0.338 | 0.22 | 0.645 | 10.42 | **0.007** |
|  | DIR × INS | 1.58 | 0.232 | 2.77 | 0.122 | 1.23 | 0.288 | 0.07 | 0.803 | 1.17 | 0.301 | 1.83 | 0.201 | 2.75 | 0.123 | 0.07 | 0.791 | 1.25 | 0.286 | 2.52 | 0.139 | 0.00 | 0.961 | 4.16 | 0.064 |
| **ESr** | DIR | 6.27 | **0.028** | 0.99 | 0.338 | 0.45 | 0.516 | 2.74 | 0.124 | 2.72 | 0.125 | 1.12 | 0.311 | 1.37 | 0.264 | 0.17 | 0.685 | 2.61 | 0.132 | 18.98 | **0.001** | 26.87 | **<0.001** | 4.25 | 0.061 |
|  | INS | 0.08 | 0.784 | 0.49 | 0.498 | 0.01 | 0.938 | 0.34 | 0.568 | 0.96 | 0.347 | 2.13 | 0.170 | 0.18 | 0.675 | 0.62 | 0.446 | 0.00 | 0.969 | 0.04 | 0.842 | 0.06 | 0.810 | 0.20 | 0.661 |
|  | DIR × INS | 0.19 | 0.672 | 0.28 | 0.609 | 0.67 | 0.429 | 0.23 | 0.637 | 0.21 | 0.656 | 0.58 | 0.462 | 0.02 | 0.880 | 0.90 | 0.362 | 0.27 | 0.611 | 0.20 | 0.666 | 0.01 | 0.907 | 1.53 | 0.239 |
| **ESl** | DIR | 0.09 | 0.767 | 0.12 | 0.733 | 0.68 | 0.425 | 1.83 | 0.201 | 0.27 | 0.611 | 1.05 | 0.326 | 1.04 | 0.327 | 1.63 | 0.226 | 11.57 | **0.005** | 33.21 | **<0.001** | 58.26 | **<0.001** | 38.65 | **<0.001** |
|  | INS | 0.01 | 0.927 | 5.55 | **0.036** | 0.10 | 0.763 | 1.09 | 0.316 | 0.57 | 0.465 | 2.05 | 0.178 | 2.76 | 0.122 | 4.47 | 0.056 | 0.03 | 0.859 | 0.02 | 0.896 | 1.10 | 0.315 | 0.05 | 0.826 |
|  | DIR × INS | 2.33 | 0.153 | 0.18 | 0.677 | 0.01 | 0.910 | 0.11 | 0.751 | 0.34 | 0.570 | 2.41 | 0.146 | 1.34 | 0.270 | 0.29 | 0.598 | 1.67 | 0.220 | 0.84 | 0.377 | 1.51 | 0.242 | 0.00 | 0.991 |
| **PEC** | DIR | 0.75 | 0.403 | 0.15 | 0.708 | 0.27 | 0.612 | 0.33 | 0.577 | 0.07 | 0.794 | 0.37 | 0.557 | 0.20 | 0.662 | 0.08 | 0.788 | 4.14 | 0.065 | 10.83 | **0.006** | 8.36 | **0.014** | 13.34 | **0.003** |
|  | INS | 2.83 | 0.118 | 3.05 | 0.106 | 0.00 | 0.971 | 0.11 | 0.742 | 1.95 | 0.188 | 0.11 | 0.750 | 0.01 | 0.943 | 0.02 | 0.900 | 0.24 | 0.635 | 0.02 | 0.897 | 0.68 | 0.426 | 0.70 | 0.419 |
|  | DIR × INS | 1.11 | 0.312 | 0.99 | 0.339 | 8.02 | **0.015** | 14.52 | **0.002** | 8.98 | **0.011** | 9.92 | **0.008** | 5.69 | **0.034** | 0.78 | 0.395 | 3.55 | 0.084 | 1.86 | 0.197 | 0.50 | 0.492 | 0.00 | 0.973 |
| **UT** | DIR | 4.03 | 0.068 | 2.73 | 0.124 | 0.26 | 0.617 | 0.24 | 0.633 | 0.05 | 0.824 | 0.08 | 0.787 | 1.18 | 0.298 | 3.69 | 0.079 | 10.70 | **0.007** | 5.04 | **0.044** | 12.14 | **0.005** | 10.73 | **0.007** |
|  | INS | 0.01 | 0.931 | 1.11 | 0.313 | 2.00 | 0.183 | 0.12 | 0.730 | 1.53 | 0.240 | 6.31 | **0.027** | 2.07 | 0.175 | 1.68 | 0.219 | 0.58 | 0.463 | 0.70 | 0.418 | 0.53 | 0.482 | 0.87 | 0.371 |
|  | DIR × INS | 0.32 | 0.584 | 0.31 | 0.588 | 0.00 | 0.950 | 6.26 | **0.028** | 0.31 | 0.587 | 0.23 | 0.638 | 0.04 | 0.854 | 1.08 | 0.320 | 1.40 | 0.260 | 0.25 | 0.626 | 0.37 | 0.556 | 0.27 | 0.615 |
| **LAT** | DIR | 1.97 | 0.186 | 0.91 | 0.358 | 0.82 | 0.382 | 0.27 | 0.615 | 0.06 | 0.813 | 1.49 | 0.246 | 1.13 | 0.309 | 1.98 | 0.185 | 3.09 | 0.104 | 11.67 | **0.005** | 19.54 | **0.001** | 8.58 | **0.013** |
|  | INS | 0.68 | 0.425 | 1.08 | 0.320 | 0.00 | 0.945 | 0.50 | 0.494 | 1.15 | 0.304 | 0.57 | 0.465 | 0.89 | 0.364 | 0.61 | 0.449 | 0.24 | 0.634 | 1.27 | 0.282 | 0.72 | 0.414 | 0.30 | 0.595 |
|  | DIR × INS | 2.36 | 0.151 | 1.60 | 0.230 | 0.00 | 0.984 | 0.59 | 0.456 | 1.00 | 0.337 | 1.00 | 0.337 | 0.73 | 0.411 | 0.04 | 0.853 | 0.23 | 0.641 | 0.02 | 0.885 | 0.02 | 0.893 | 2.66 | 0.129 |
| **SER** | DIR | 1.40 | 0.259 | 0.00 | 0.996 | 5.29 | **0.040** | 4.31 | 0.060 | 0.01 | 0.929 | 2.04 | 0.179 | 1.38 | 0.263 | 0.53 | 0.482 | 14.95 | **0.002** | 20.78 | **0.001** | 12.85 | **0.004** | 11.61 | **0.005** |
|  | INS | 0.25 | 0.625 | 0.30 | 0.597 | 0.72 | 0.413 | 3.68 | 0.079 | 3.49 | 0.086 | 0.11 | 0.747 | 0.07 | 0.800 | 1.02 | 0.333 | 0.44 | 0.521 | 3.28 | 0.095 | 2.35 | 0.152 | 0.99 | 0.338 |
|  | DIR × INS | 0.03 | 0.877 | 0.85 | 0.376 | 0.04 | 0.852 | 0.38 | 0.548 | 6.05 | **0.030** | 2.81 | 0.120 | 3.80 | 0.075 | 1.94 | 0.189 | 0.12 | 0.734 | 0.78 | 0.394 | 0.01 | 0.930 | 0.25 | 0.627 |

Legend: DIR – Direction, INS – Instruction, DIR × INS – interaction between Direction and Instruction
